# Dissecting acute neuronal responses to glioblastoma using a dual-interface human iPSC neuronal culture platform

**DOI:** 10.1186/s40478-026-02312-z

**Published:** 2026-05-16

**Authors:** Ouada Nebie, Niyi Adelakun, Brian Fries, Luke Kollin, Liwen Zhang, Akhil Medikonda, Monica Venere, Pierre Giglio, Nam Chu, Nhat Le

**Affiliations:** 1 Department of Cancer Biology and Genetics, College of Medicine, The Ohio State University Wexner Medical Center, Columbus, OH 43210 USA; 2 Comprehensive Cancer Center, The Ohio State University Wexner Medical Center, Columbus, OH 43210 USA; 3 The Ohio State Biochemistry Program (OSBP), The Ohio State University, Columbus, OH 43210 USA; 4 Campus Chemical Instrument Center, Mass Spectrometry and Proteomics, The Ohio State University, Columbus, OH 43210 USA; 5 Department of Radiation Oncology, The Ohio State University Comprehensive Cancer Center, Columbus, OH 43210 USA; 6 Department of Neurology, College of Medicine, The Ohio State University Comprehensive Cancer Center, The Ohio State University Wexner Medical Center, Columbus, OH 43210 USA; 7 Solove Research Institute Comprehensive Cancer Center, College of Medicine, James Cancer Hospital, The Ohio State University Comprehensive Cancer Center, The Ohio State University Wexner Medical Center, Columbus, OH 43210 USA

**Keywords:** Glioblastoma, iPSC-derived neurons, Tumor-neuron interaction, MAPK signaling, ERK1/2, p38α MAPK, Synaptic remodeling, Patient-derived glioblastoma cells

## Abstract

Glioblastoma (GB) hijacks neuronal circuits to promote tumor progression, but the earliest neuronal responses remain poorly defined. We developed a dual-interface human iPSC-derived neuronal model to study acute paracrine signaling triggered by GB cells from two sources: serum-adapted U-87MG and serum-free NU-757. Within 24 h, exposed neurons displayed synaptic remodeling and activation of GB-related signaling cascades. Neurons exposed to U-87MG showed decreased dendritic spine number alongside increased total ERK and phospho-p38α at spines (1). In the soma, total ERK accumulated in the nucleus while phospho-ERK was primarily cytoplasmic; nuclear p38 and cytoplasmic MLK2 also increased (2). Conversely, NU-757 exposure enhanced spine growth but reduced postsynaptic density, NMDAR, and synaptophysin levels (3). Both total and phospho-ERK showed increased nuclear localization with NU-757, while total MLK2 and p38α levels remained stable but exhibited elevated nuclear (4) and spine localization. Pharmacological MEK/ERK inhibition reduced U-87MG proliferation and migration and restored neuronal spine numbers. This model system reveals source-dependent, compartment-specific signaling dynamics that govern synaptic vulnerability and provides a platform for investigating GB initiation, recurrence, and progression, as well as therapies targeting tumor growth and neural circuit remodeling.

**Graphical abstract:**

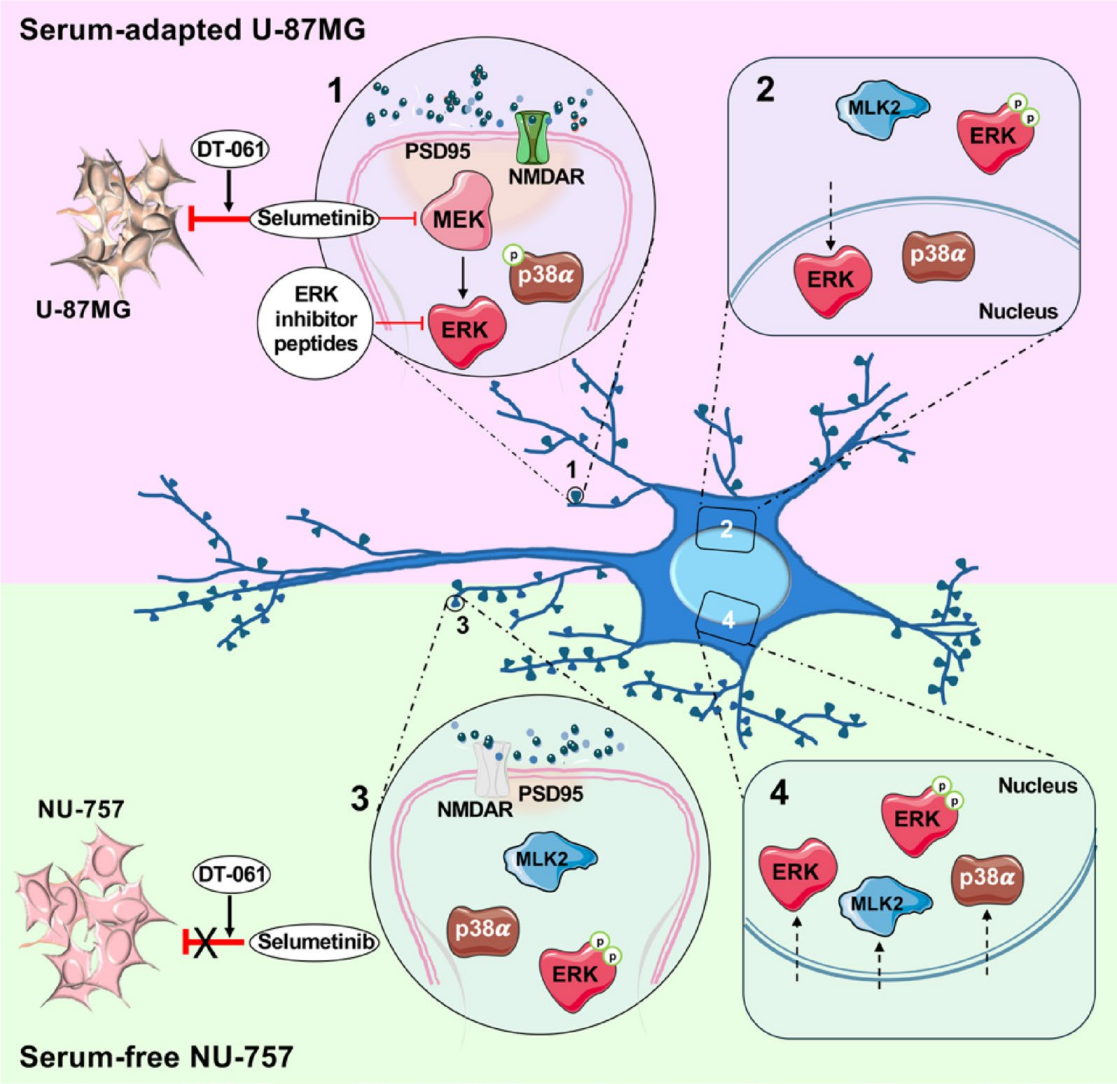

**Supplementary Information:**

The online version contains supplementary material available at 10.1186/s40478-026-02312-z.

## Introduction

Gliomas are primary brain tumors, with glioblastoma (GB) representing the most aggressive and lethal subtype. Despite advances in surgery, radiotherapy, and chemotherapy, GB remains uniformly fatal, with a median survival of just 15 months [1–3]. Recurrence, rather than initial tumor growth, is the ultimate cause of death, yet its biological drivers remain unknown [4]. This poor outcome reflects not only the tumor’s resistance to therapy but also its ability to remodel the brain microenvironment, including neighboring neurons, to facilitate growth and invasion [5, 6].

Recent studies have revealed that neuronal activity actively contributes to glioma progression. Glioma cells form synaptic-like connections with neurons [7–10] and co-opt excitatory neurotransmission to drive tumor growth and treatment resistance [11, 12]. These interactions also disrupt normal neuronal function, enhancing excitotoxicity and altering circuit dynamics [13]. Paradoxically, some standard therapies, including radiation and anti-angiogenic agents, may further promote tumor cell infiltration into neighboring nonneoplastic brain parenchyma [14, 15]. However, modulating neuronal activity has been shown to slow tumor growth [16–18], underscoring the importance of understanding GB-neuron interactions early in the disease progression.

Despite this emerging interest, mechanistic insight into how GB cells influence human neurons during initial tumor infiltration remains limited. Existing animal models and human in vitro systems lack the resolution or physiological relevance needed to examine acute, spatially defined tumor-neuron dynamics [19, 20]. Additionally, most therapeutic developments still focus primarily on tumor-intrinsic targets, often overlooking the tumor-neuron interface, which is increasingly recognized as a driver of disease progression and therapeutic resistance [13, 21–24].

To address this, we created a co-culture platform derived from human iPSCs to investigate early interactions between GB cells and neurons. We used both the serum-adapted U-87MG cells, a widely used GB cell line, and a serum-free patient-derived GB line NU-757 that more closely reflects human GB heterogeneity. We discovered that GB exposure promptly triggers synaptic remodeling and adapts to GB signature proteomes. Exposure of neurons to U-87MG cells rapidly triggered activation of MAPK signaling, driving neurons toward a tumor promoting state. MEK inhibition protected neuronal integrity while suppressing U-87MG proliferation and invasion. In contrast, neurons exposed to the patient-derived NU-757 cells showed a distinct dynamic signaling and synaptic remodeling profile, compared with U-87MG, underscoring heterogeneity among tumor sources. This model allows for high-resolution investigation of tumor-neuron interactions and highlights the potential differentiation of individual GB heterogeneity/sources.

Our streamlined human model system is innovative in focusing on the earliest molecular and circuit‑level interactions between neurons and GB, a window that has been largely overlooked in favor of advanced disease. By reframing neurons as active, programmable components of the tumor microenvironment and by directly targeting neuron‑intrinsic signaling, the work introduces a potential therapeutic paradigm that goes beyond tumor‑centric strategies. The concepts and platform developed are broadly applicable to brain metastases and other neuroinvasive cancers, establishing generalizable principles for neuron-tumor communication.

## Results

### A human dual-interface co-culture system to study early neuronal response to glioblastoma

**Fig. 1:**
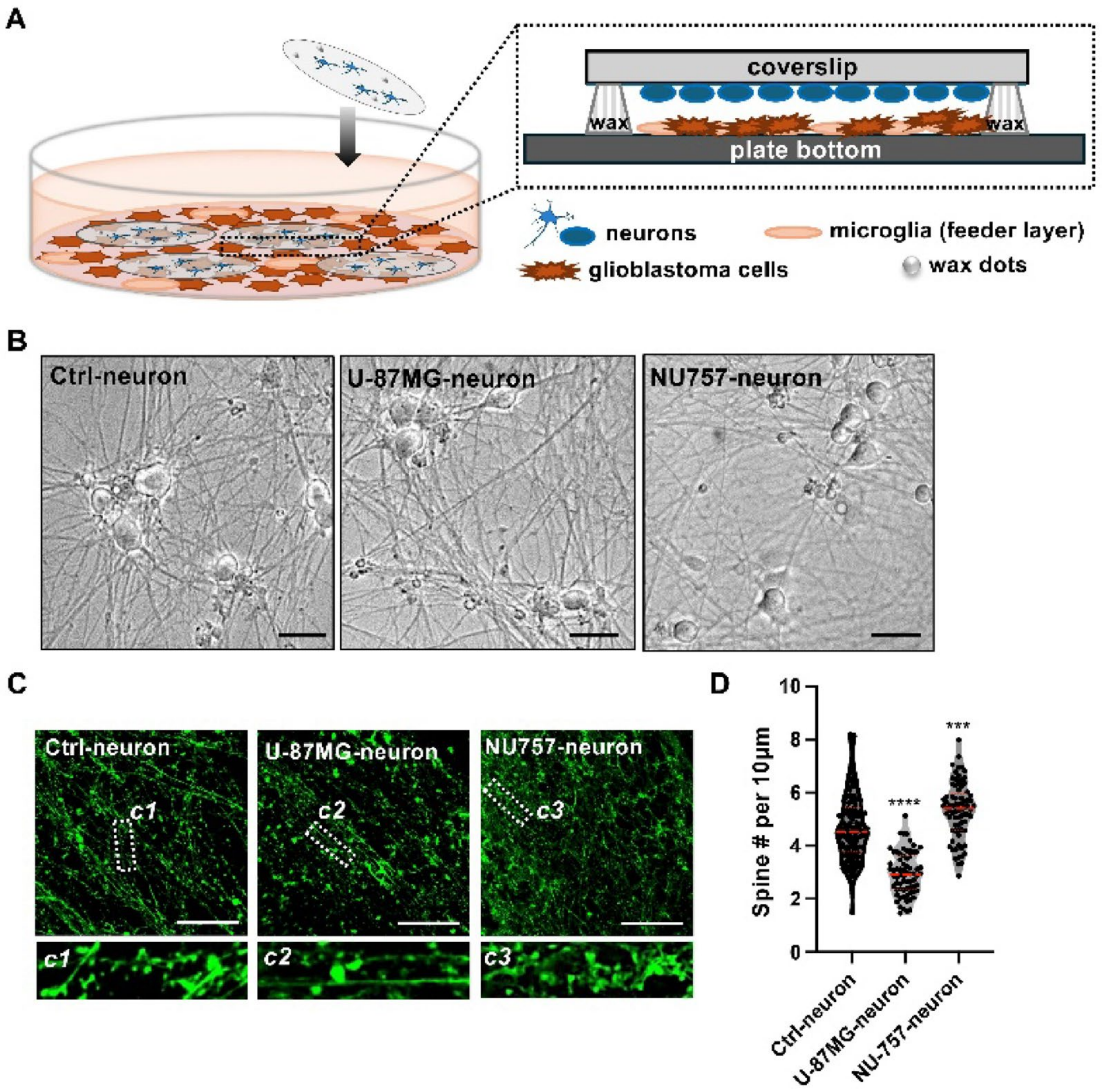
A dual-interface human glioblastoma-neuronal co-culture system reveals glioblastoma-induced changes in neuronal morphology. **A** Schematic of the dual-interface human glioblastoma (GB)-neuronal co-culture system used to study acute neuronal responses to GB. Human iPSC-derived neurons are cultured on glass coverslips suspended approximately 0.5 mm above a layer of human microglial cells using wax dots as spacers. GB cells are introduced into the microglial layer beneath the neurons. **B** Representative differential interference contrast (DIC) images of human iPSC-derived neurons cultured under three conditions: control (Ctrl-neuron), exposed to U-87MG cells (U-87MG-neuron), or exposed to serum-free patient-derived NU-757 cells (NU-757-neuron) at 24 h. Scale bars = 30 μm. **C** Fluorescent phalloidin staining (green) of dendritic spines in neurons after 35 days of differentiation under the same conditions as in (B). Insets (c1, c2, c3) show magnified views of the dashed regions. Scale bars = 50 μm. **D** Quantification of dendritic spine density (spines per 10 μm) corresponding to (**C**). Data were pooled from 70–160 dendritic segments across 2–3 independent experiments. U-87MG exposure significantly reduced spine density, whereas NU-757 exposure led to increased spine density compared to controls. Data are shown as mean ± SD. Statistical comparisons were performed using unpaired t-tests. Significance: ***P < 0.001; ****P < 0.0001

To investigate the early molecular and functional effects of GB on human neurons, we established a dual-interface co-culture system using GB cells and human iPSC-derived cortical neurons (Fig. 1A). This system was specifically designed to model the acute phase of tumor-neuron interactions and address some limitations of static, non-compartmentalized culture systems. Unlike traditional organoids or mixed co-cultures that lack spatial precision, our approach allows direct yet controllable exposure of neurons to GB cells, enabling the identification of rapid, cell-type-specific changes at the tumor-neuron interface.

We employed a neuronal differentiation protocol [25, 41] in which human iPSCs (hiPSCs) can be induced to differentiated cortical neurons in an efficient and scalable manner. Human iPSCs were differentiated into excitatory cortical neurons over 3–4 weeks and matured in monolayer culture on coated coverslips. Neuronal identity and synaptic maturity [26] were validated through expression of MAP2, synaptophysin, and PSD-95 (Fig. S1). Neurons differentiated from hiPSCs develop functionally active synapses [25]. On day 35 post-differentiation, neurons were suspended above a human microglia layer and maintained in serum-free medium. GB cells were introduced into the system by being seeded onto the bottom microglia layer to allow molecular exchange without overgrowth (Fig. 1A, B). This configuration enabled acute co-culture periods, allowing GB-derived factors to influence neurons before irreversible structural changes or tumor overtake occurred.

To ensure the system maintained biological relevance during the acute exposure window, we confirmed neuronal viability and morphologies in control conditions without exposure to GB. Neurons in these cultures, which are maintained up to weeks in the presence of a human microglia cell layer, keep morphologically matured axons and dendrites (Fig. 1B, Ctrl-neuron), and functional excitatory and inhibitory synapses [27]. The dendrites are studded with a high density of mushroom-shaped and stubby spines, which can be stained with fluorescently labeled phalloidin by virtue of its ability to bind to actin filaments within the spines (Fig. 1C, Ctrl-neuron). Notably, GB cells retained viability (Fig. S7A) and invasive potential in the interface conditions, consistent with features associated with early tumor infiltration [28].

### Immortal serum-adapted U-87MG but not serum-free patient-derived NU-757 glioblastoma cell lines induces changes in synaptic morphologies

Relying on the dual-interface co-culture system described in Fig. 1, we next investigated whether acute GB cell exposure affects neuronal connectivity and synaptic organization in human cortical neurons. This platform, which was designed to preserve spatial fidelity and allow controlled, non-overlapping interaction between differentiated human neurons and GB cells, provided a unique opportunity to study early, cell-type-specific effects on synaptic morphology before irreversible structural alteration occurs.

To assess whether the system could distinguish neuronal responses to different GB sources, we compared the effects of two GB sources: the commonly used serum-adapted U-87MG cell line [29] and the serum-free patient-derived NU-757 line [30], both introduced at the base of the microglia-supported interface. Human iPSC-derived cortical neurons at day 35 of differentiation, featuring mature dendritic arbors and high spine density (Fig. 1B, C, Ctrl-neuron), were exposed to GB cultures over a 24-hour period. Importantly, the use of a human microglia layer in the interface did not compromise the cell viability or neuronal structural integrity, providing a context that permits paracrine signaling interactions relevant to early tumor-neuron communication.

Neurons co-cultured with U-87MG cells exhibited marked alterations in dendritic spine morphology. Phalloidin staining showed a loss of mature spine types, particularly mushroom-shaped spines, suggesting cytoskeletal remodeling at excitatory synapses. In contrast, exposure to the serum-free cell line cultured from patient tumor tissue NU-757 did not induce significant alterations in synaptic morphology, but an increase of spine number compared to control cultures (Fig. 1C, D, sample sizes: 70–150 neurite locations). Alongside changes in spine numbers, Western blot analysis revealed differential expression of synaptic proteins, including presynaptic synaptophysin and postsynaptic PSD-95 and NMDAR2B, between the two GB models (Fig. S1). Whereas neurons exposed to U-87MG presented only a significant downregulation of PSD-95 proteins, the expression of all three proteins was significantly reduced in those exposed to NU-757 GB cells. These differences likely reflect distinct modes of tumor-neuron interaction rather than differences in intrinsic tumor aggressiveness, highlighting how variant these two GB sources can elicit distinct neuronal responses within the tumor microenvironment.

These findings underscore both the sensitivity and versatility of our co-culture system in capturing subtle effects on neuronal morphology and synaptic markers.

### Acute glioblastoma exposure triggers disease-associated proteomic signatures in healthy human neurons

Given the pronounced synaptic remodeling observed following acute exposure to GB cells, we next asked whether these synaptic alterations were accompanied by an acute global shift in protein expression in neurons. Using our dual-interface co-culture model, we performed an unbiased proteomic analysis to capture neuronal responses at the protein level during this critical early window of tumor-neuron interaction.

To isolate neuron-specific proteomic changes, human iPSC-derived cortical neurons were cultured normally (control) or exposed for 24 h to either U-87MG or NU-757 GB cells in the dual-interface system (Fig. 1). Following exposure, neuronal cultures were carefully dissociated from the upper layer, excluding GB and microglial lower layers, and were subjected to label‑free quantitative proteomic analysis using data-dependent acquisition (DDA). Across conditions, over 5,500 proteins were reliably quantified (Fig. S2).

Neurons exposed to U-87MG cells exhibited a robust proteomic shift relative to controls, with 520 proteins significantly altered (adjusted *p* < 0.05, fold change > 1.5).

In sharp contrast, neurons exposed to patient-derived NU-757 glioblastoma cells showed a much more constrained proteomic response. Only 83 proteins were differentially expressed compared to controls.

**Fig. 2:**
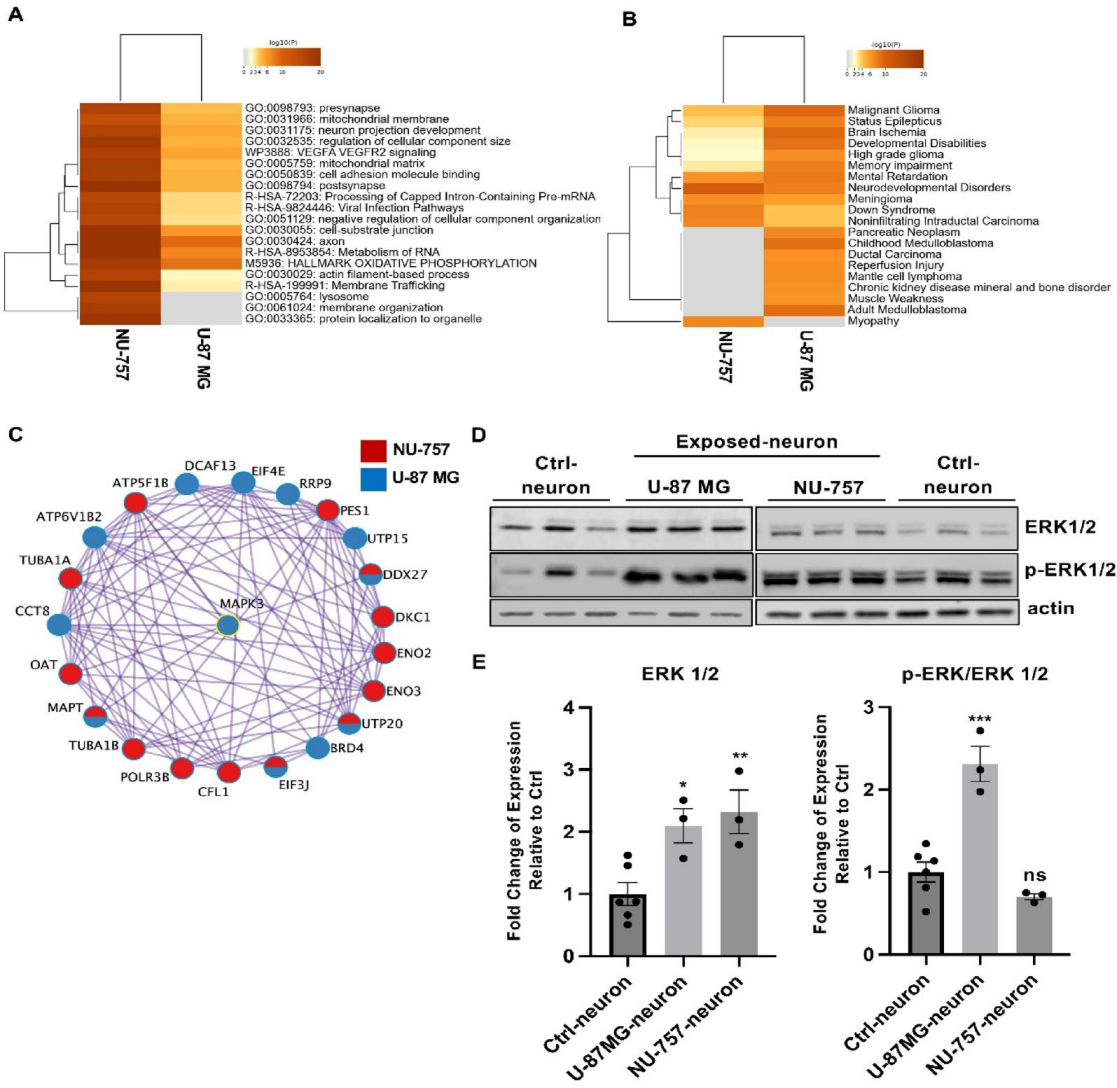
Proteomic analyses reveal distinct signaling pathway activation in neurons exposed to different glioblastoma cells. **A** Heatmap showing pathway and process enrichment analysis of differentially expressed proteins in neurons exposed to NU-757 versus U-87MG glioblastoma cells for 24 h. Enrichment was performed using described ontology sources. Heatmap cells are color-coded by* p*-value; white cells indicate no significant enrichment for that term in the respective protein set. **B** Heatmap of DisGeNET disease enrichment analysis based on differentially expressed neuronal proteins after 24-hour exposure to NU-757 versus U-87MG glioblastoma cells. Heatmap cells are colored by* p*-value, with white cells indicating no enrichment. **C** Protein-protein interaction (PPI) network centered on ERK1/2 (MAPK3), highlighting significantly altered proteins in neurons exposed to NU-757 (red) or U-87MG (blue) glioblastoma. **D** Immunoblot analysis of neuronal lysates from dual-interface co-cultures, including control neurons (Ctrl-neuron), neurons exposed to U-87MG (U-87MG-neuron), and neurons exposed to NU-757 (NU-757-neuron). Blots were probed for total ERK1/2, phosphorylated ERK1/2 (p-ERK1/2), and actin as a loading control. Data represent three independent experiments (*n* = 3). **E** Quantification of ERK1/2 and its phosphorylated form, p-ERK1/2 immunoreactivity in the blots shown in D. Data shown is the mean ± SEM. Statistical analysis was performed using unpaired t-tests; Significance is indicated as: ns (not significant), *P < 0.05, **P < 0.01, ***P < 0.001

Gene Ontology (GO) and pathway enrichment analyses of the proteomic data from neurons exposed to U-87 and NU-757 glioblastoma cells revealed significant changes in key neuronal structural and functional pathways (Fig. 2A, S3A and B). Both models showed enrichment in terms related to synaptic organization, including presynaptic and postsynaptic compartments, axonal structure, cell junctions, and neuron projection development (Fig. 2A). These pathways collectively point to alterations in pathways related to neuronal connectivity and synaptic organization, suggesting that GB exposure affects molecular pathways associated with neuronal network organization. This molecular evidence aligns with our synaptic structural observations: neurons exposed to U-87MG cells exhibited reduced MAP2 expression (Fig. S1C) and dendritic spine loss (Fig. 1C), while those exposed to NU-757 retained more typical neuronal architecture. Interestingly, despite these differences in structural responses, both tumor models induced proteomic signatures associated with high-grade and malignant gliomas, as revealed by DisGeNET analysis (Fig. 2B). This suggests that the GB microenvironment, regardless of the source of GB cells, triggers a shared set of disease-relevant molecular changes in neurons, particularly targeting pathways essential for maintaining synaptic integrity and neuronal projection. These findings suggest shared molecular responses by which GB exposure perturbs neuronal pathways, with potential implications for both tumor progression and neurological decline in affected patients. Importantly, this finding highlights the capacity of our co-culture system to detect the molecular consequences of tumor exposure in a cell-type-specific and time-sensitive manner.

In sum, these data suggest that early exposure to GB-derived signals can trigger disease-associated molecular responses in neurons, potentially priming the brain microenvironment for long-term dysfunction. The integration of proteomic profiling into our model offers a valuable readout for mechanistic investigation and therapeutic targeting of tumor-neuron communication.

### Glioblastoma induces activation and alters subcellular distribution of MEK/ERK pathways in peritumor neurons

**Fig. 3:**
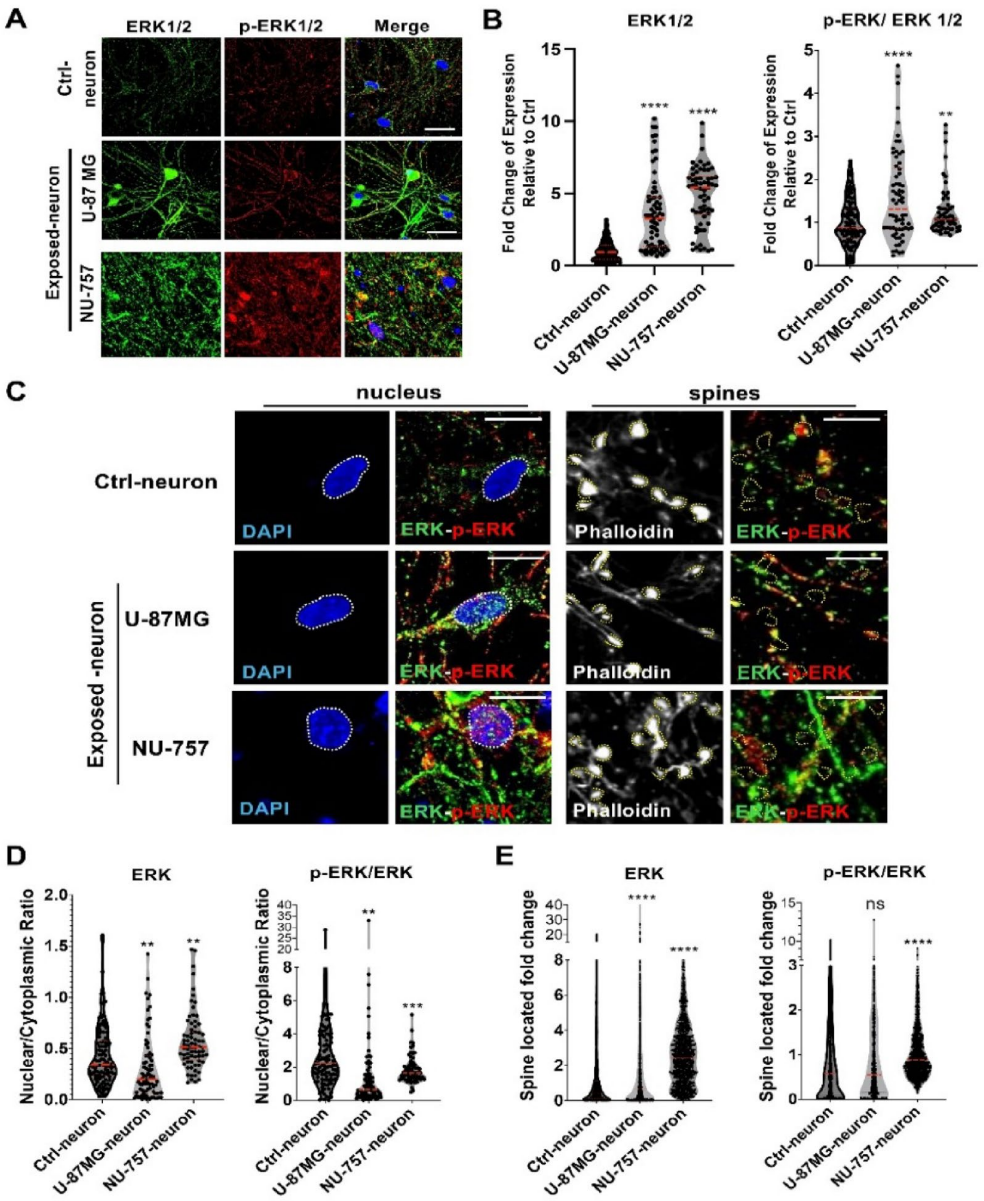
Differential subcellular localization and alteration of ERK1/2 signaling in neurons exposed to glioblastoma cells. **A** Representative immunofluorescence images showing total ERK1/2 (green) and phosphorylated ERK1/2 (p-ERK1/2, red) in neurons exposed for 24 h to U-87MG or NU-757 glioblastoma cells, compared to controls. Nuclei were stained with DAPI (blue). Scale bars = 50 μm. **B** Quantification of ERK1/2 immunofluorescence intensity in neuronal cultures exposed to U-87MG or NU-757 glioblastoma for 24 h versus controls. Data represent pooled measurements from 62–102 randomly selected neuronal fields per independent experiment. Values are mean ± SD. Statistical analysis was performed using unpaired t-tests; significance: ****P < 0.0001. **C** Representative images of triple immunofluorescence staining for phalloidin (gray), total ERK1/2 (green), and p-ERK1/2 (red) highlighting different neuronal compartments after 24-hour glioblastoma exposure. Dotted lines outline nuclei (DAPI, blue) and intact dendritic spines as indicated by phalloidin staining. Scale bars = 20 μm and 5 μm for nucleus panels and spine panels, respectively. **D–E** Quantification of ERK1/2 immunofluorescence intensities in distinct neuronal compartments from images in (**C**). **D** Ratio of ERK1/2 levels in nucleus versus cytoplasm, pooled from 72–111 locations across three independent experiments. (E) ERK1/2 levels at spine locations, based on 708–2186 measurements from three independent experiments. Data are presented as mean ± SD. Statistical comparisons used unpaired t-tests. Significance is indicated as: ns (not significant), **P < 0.01, ***P < 0.001, ****P < 0.0001

To elucidate the intracellular pathways underpinning GB-induced synaptic and molecular alterations, we focused on the MEK/ERK cascade, a critical mediator of neuronal stress responses and synaptic remodeling. Building on proteomic pathway enrichment (Fig. 2C), we performed immunoassays on iPSC-derived cortical neurons after 24 h of GB exposures. Western blot (WB) (Fig. 2D) and immunofluorescence (IF) (Fig. 3A, C) analyses show significant changes in both ERK1/2 total levels and phosphorylation levels of ERK1/2 (p-ERK1/2) in GB-exposed neurons. High-resolution IF to localize these proteins in neurons show that U-87MG exposure exhibited significantly elevated p-ERK levels in different neuron compartments, in the nucleus and soma but not dendrites, with no change (*p* = 0.7, sample size: 1102–2186 locations) in spine-localized p-ERK puncta (Fig. 3C, E) and a ~ 4.2-fold decrease (*p* < 0.0001, sample size: 81–111 locations) in nuclear/cytoplasm ratio of p-ERK intensity (Fig. 3C, E) compared to controls. In contrast, neurons exposed to patient-derived NU-757 cells showed no significant changes in p-ERK nuclear/cytoplasm ratio (*p* = 0.06, sample size: 72–111 locations) (Fig. 3C, D), but ~ 4 times increase (*p* < 0.0001, sample sizes: 1349–2186 locations) at spines location (Fig. 3C, E), consistent with their preserved synaptic morphology distinct from U-87MG (Fig. 1C). These findings indicate that GB-derived signals can differentially engage spatially distinct ERK signaling in neurons, suggesting that individual tumor sources elicit unique modes of neuronal response.

Expanding this analysis, we assessed additional MAPK family members, mixed lineage kinase 2 (MLK2) and p38 MAPK, both of which are implicated in regulating cytoskeletal dynamics and dendritic spine morphology [31]. MLK2 is significantly upregulated in the proteomes of U-87MG-exposed neurons (Fig. S3C, MAP3K10). As an upstream kinase that activates MAPK signaling pathways, including JNK and p38, MAPK signaling has been implicated in regulating actin cytoskeleton remodeling and dendritic spine stability, while p38 MAPK modulates synaptic plasticity and spine pruning through phosphorylation of cytoskeletal-associated proteins. WB analysis of GB-exposed neurons shows MLK2 and phospho p38α levels significantly upregulated in U-87 exposure but not in NU-757 exposure compared to control neuron samples (Fig. S4A, B). Interestingly, the following analysis of subcellular distribution of these proteins in neurons provided another informative perspective. Neurons exposed to NU-757 cells showed a significant increase in nucleus-localized MLK2 level (~ 1.6-fold) and p38α level (~ 1.2-fold) relative to controls (Fig. S4C–E). This elevated nuclear MLK2 level is in contrast with neurons exposed to U-87MG cells, where MLK2 remained at baseline for both nucleus and spine locations (Fig. S4C). At spine locations, neurons exposed to NU-757 cells showed a significant increase for both MLK2 (~ 2.0-fold), and p38α (~ 1.9-fold). Neurons exposed to U-87MG cells showed a significant increase in p38α (~ 1.3-fold). This differential activation correlates with the distinct synaptic phenotypes observed: U-87MG exposure induces spines loss and aberrant morphology, while NU-757 exposure preserves spine density and structure (Fig. 1C).

The selective upregulation of the p38 family in U-87MG-exposed neurons in both nucleus and spine locations likely contributes to destabilization of actin filaments and dendritic spines, as these kinases are known to phosphorylate cytoskeletal regulators such as MAP2 and PSD95, leading to spine shrinkage and synaptic weakening. Conversely, the absence of this activation in nucleus locations of NU-757 conditions may explain the maintenance of normal spine morphology despite tumor exposure.

Together, these findings underscore the unique capacity of our dual-interface co-culture model (Fig. 1A) not only to discriminate GB cell-specific effects on neurons but also to resolve signaling events with subcellular precision, capturing dynamic and compartmentalized neuronal responses in both acute and potentially chronic tumor microenvironments.

### MEK pathway inhibition potentially reverses spine loss in U-87MG-exposed neurons and impairs glioblastoma growth and migration

**Fig. 4:**
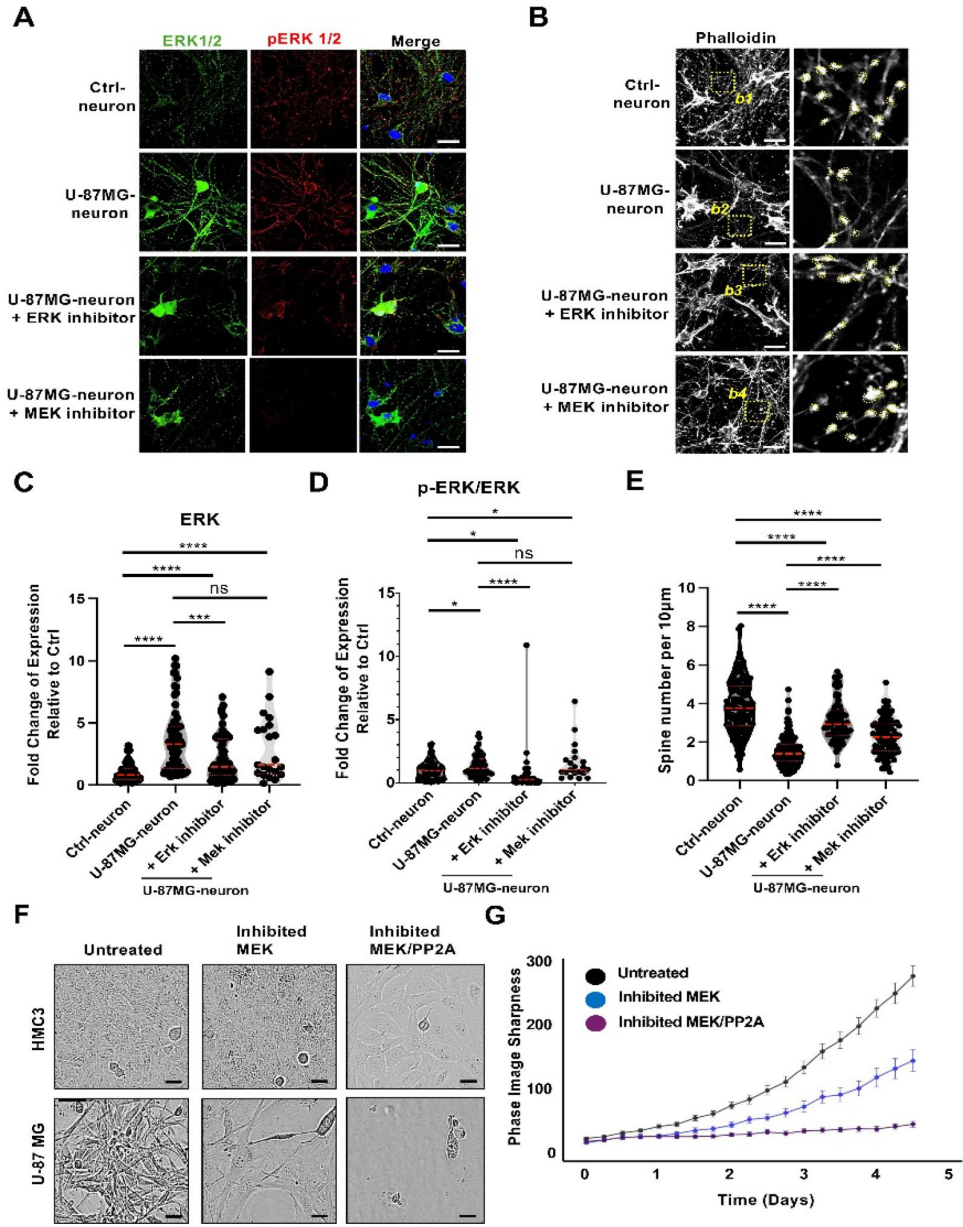
MEK/ERK signaling inhibition reduces glioblastoma-induced neuronal ERK activation and spine loss. **A** Representative immunofluorescence images of total ERK1/2 (green) and phosphorylated ERK1/2 (p-ERK1/2, red) in neurons under different conditions: control (Ctrl-neuron), exposed to U-87MG glioblastoma cells (U-87MG-neuron), U-87MG-exposed neurons treated with an ERK peptide inhibitor (U-87MG-neuron + ERK inhibitor), or U-87MG-exposed neurons treated with the MEK inhibitor Selumetinib (U-87MG-neuron + MEK inhibitor). Nuclei were stained with DAPI (blue). Scale bars = 30 μm. **B** Fluorescent phalloidin staining (gray) of dendritic spines in neurons after 35 days of differentiation under the same conditions as in (**A**). Insets (b1–b4) show magnified views of the dashed regions. Scale bars = 30 μm. **C–D** Quantification of immunofluorescence intensity of total ERK1/2 (**C**) and p-ERK1/2 (**D**) in neurons under each condition from (**B**). Data are pooled from 21–67 randomly selected neuronal fields per independent experiment. Values represent mean ± SD. Statistical comparisons were performed using unpaired t-tests. Significance: ns (not significant), *P < 0.05, ****P < 0.0001. **E** Quantification of dendritic spine density (spines per 10 μm) under each condition from (**B**). Data represent 72–160 dendritic segments from 2–3 independent experiments. Values are shown as mean ± SD. Unpaired t-tests were used for statistical analysis; ****P < 0.0001. **F–G** Phase-contrast images (**F**) and quantification (**G**) of HMC3 microglia and U-87MG cells imaged using Incucyte. Treatment with Selumetinib (MEK inhibitor) or Selumetinib combined with DT-061 (MEK/PP2A pathway inhibition) reduced U-87MG cell proliferation and migration, and induced cell death. Scale bars = 30 μm. Values are shown as mean ± SEM. Data are pooled from 4 cultures per each condition; 5 fields were imaged per each culture

Having established that U-87MG glioblastoma cells induce MEK/ERK-dependent signaling in neurons exposed to them (Figs. 2 and 3), we next tested whether pharmacological inhibition of this pathway could partially restore dendritic spine morphology and impair GB cell migration/invasion. Human iPSC-derived cortical neurons were treated with the MEK1/2 inhibitor Selumetinib [32], an FDA-approved drug for neurofibromatosis type 1 (NF1), or an ERK inhibition peptide [33], during the 24-hour co-culture period with U-87MG cells in the dual-interface system. These MEK and ERK inhibitions partially restored neuronal morphology at the dendritic spines level. Phalloidin staining of actin-rich dendritic spines revealed a significant increase of spine density, (Fig. 4B, E). Quantitative imaging confirmed suppression of phosphorylated ERK (p-ERK) in neurons, validating effective MEK pathway blockade (Fig. 4A, C, D).

Under the same conditions, U-87MG GB cells exhibited significantly impaired expansion and migration. MEK inhibition by Selumetinib reduced the two-dimensional surface coverage of U-87MG cells by ~ 55% (*p* < 0.001) and blocked their transwell invasiveness through the ECM-coated membrane (Fig. 4F, G and Fig. S5C). The viability of the treated cells was not affected, suggesting that MEK inhibition alone does not induce cytotoxicity.

To identify complementary targets that might amplify the anti-tumor effect, we examined our proteomic data (Sect. 3, Fig S5A) for neuronally regulated factors with tumor-suppressive potential. Notably, Protein Phosphatase 2 A (PP2A) regulatory subunits were downregulated in neurons exposed to U-87MG (Fig. S5B), suggesting a GB-driven suppression of this critical phosphatase. Given PP2A’s known role in antagonizing oncogenic MAPK signaling and stabilizing cytoskeletal architecture, we hypothesized that PP2A activation could synergize with MEK inhibition.

Treatment with the small-molecule PP2A activator DT-06 [34] alone had exerted minimal effects on U-87MG cell proliferation and migration, except 10 µM concentration of treatment (Fig S6A). However, a combination of DT-061 and Selumetinib suppressed GB cells expansion and trans-well migration, and notably, induced cytotoxic death in U-87MG cells compared with the mock and single treatments (Fig. 4F, G and Fig. S5E, F). This synergistic effect may reflect disruption of kinase-phosphatase balance within GB cells and highlights a promising therapeutic interaction between phosphatase reactivation and kinase inhibition.

Importantly, this dual-target approach had no toxic effects on control neurons or neurons co-cultured with patient-derived NU-757 glioblastoma cells compared to serum-adapted GB U-87cells. Moreover, NU-757 cells showed no significant change in growth, migration, or viability under MEK inhibition (Fig. S5D). This differential response suggests that NU-757 cells rely less on MAPK signaling, consistence with NU-757-exposed neuronal proteome data and instead depend on distinct molecular pathways for proliferation and survival. On the other hand, the MEK, ERK inhibitors and the PP2A activator were evaluated and showed no signs of toxicity on normal healthy neurons (Fig. S6E). These findings underscore the biological heterogeneity of GB, and the importance of tailored therapeutic strategies based on tumor type and signaling context, a point we further explore in the Discussion.

## Discussion

The interaction between GB and neurons is increasingly recognized as a bidirectional process that not only shapes the progression of the tumor but also contributes to neurological dysfunction in patients [35]. Furthermore, current GB treatments fail to address the invasive aspect of tumor spread, which is responsible for recurrence and disease progression. Thus, there is an urgent need for a better understanding of tumor biology at the infiltrative margin, as well as the development of improved combination therapies that address both tumor proliferation and migration for all GB patients. In this study, we established a novel dual-interface co-culture system to model early, spatially controlled interactions between human GB cells and iPSC-derived cortical neurons. This system allowed us to uncover tumor cell-specific effects on neuronal morphology, proteomic reprogramming, and intracellular signaling with subcellular resolution, and to identify potential therapeutic targets that affect both tumor and neuronal compartments.

One of the key innovations of this platform lies in its capacity to model acute tumor-neuron interactions with defined spatial architecture, address some limitations of static organoids and mixed co-cultures. The system facilitates the separation of neurons from GB cells following exposure, enabling a precise investigation of neuron-specific pathogenic events that precede GB cell proliferation and invasion. The use of mature, synaptically active human cortical neurons layered above a microglial monolayer provides a biologically relevant context in which GB-secreted factors can influence neurons without physical overgrowth. This design enables the resolution of early molecular and functional changes that precede irreversible structural damage, offering a tractable window for intervention and mechanistic dissection.

While our current dual-interface culture system incorporates neurons, microglia, and GB cells to better approximate key cellular components of the brain tumor microenvironment, it is not intended to isolate individual pairwise signaling mechanisms. Instead, this platform is designed to capture early multicellular paracrine interactions that influence neuronal synaptic remodeling, which can subsequently be dissected in more reductionist co-culture configurations. The 24-hour exposure window used in this study was selected to capture early neuronal responses to GB-derived signals, as synaptic structural changes were reproducibly detectable within this timeframe while minimizing longer-term culture adaptation effects. The findings presented here capture early neuronal responses to tumor-derived signals rather than long-term network adaptation to tumor growth.

Using this system, we discovered that the commonly used serum-adapted GB line U-87MG and the serum free patient-derived NU-757 glioblastoma cells, both induced rapid and significant GB-derived neuronal proteome alterations and remodeling of dendritic spines. These structural and synaptic modifications occur during early tumor–neuron interactions, prior to the later functional integration and network alterations reported in other GB models. This finding is supported by Svenja K. Tetzlaff et al. [36]. They noted that in certain patient-derived models, the majority of GB cells and neurons form early functional connections during GB invasion, prior to the onset of neuronal dysfunction and hyperexcitability, which appear later in the disease [36]. The changes observed in U-87MG-exposed neuron were accompanied by a robust proteomic shift toward degenerative, inflammatory, and cytoskeletal remodeling pathways, while neurons co-cultured with NU-757 cells increased synaptic structures. This divergence underscores the importance of GB heterogeneity in shaping neuronal responses and highlights the utility of our system for modeling such subtle differences. In addition, the differential outcomes between U-87MG and NU-757 highlight the limitations of using serum adapted cell lines as generalizable GB models and emphasize the need for incorporating serum free patient-derived cells that reflect the phenotype of the tumor type of origin. It is also well documented that GB cell lines including U-87MG present discrepancies in their mutational profiles and their original tumor tissue suggesting that they have deviated from their initial characteristics after a certain amount of time in culture [37]. This could also explain the differences observed.

Our data show that neurons exposed to U-87MG, in addition to the loss of dendritic spines (Fig. 1C), exhibit reduced MAP2 expression (Fig. S1B). MAP2 is a neuronal protein that works as a dendrite and dendritic spine marker, interacting with microtubules to maintain dendritic shape [38] and its downregulation indicate structural degeneration and impaired synaptic integrity. While previous studies have highlighted the ability of GB cells to form synaptic connections with neurons, enhancing tumor growth through neuron-to-glioma glutamatergic signaling [39, 40], our findings suggest that this U-87MG neuronal interaction may come at the expense of normal neuron-neuron connectivity. The decrease spine number suggests that neuron-GB interactions serve a dual purpose: they may contribute to tumor progression while impairing neuronal integrity. The increase in actin-rich dendritic protrusions observed in neurons exposed to NU-757 by phalloidin labeling were interpreted as evidence of cytoskeletal remodeling rather than a direct measure of functional synapse formation, which may explain the concurrent reduction in several synaptic protein markers detected by immunoblotting. This highlights the complex and detrimental impact of GB on surrounding neural networks.

Mechanistically, we identified activation of the MEK/ERK signaling cascade as a important mediator of the neuronal response to U-87MG glioblastoma cells. High-resolution imaging revealed ERK activation not only in the neuronal soma and nucleus but also at dendritic spines, suggesting concurrent modulation of transcriptional programs and local synaptic plasticity. This response was further supported by selective changes of MLK2 and p38 MAPK, two additional stress-responsive kinases with known roles in cytoskeletal regulation and synaptic destabilization [31]. Notably, these pathways were not activated similarly in neurons exposed to NU-757 cells. The absence of comparable pathway activation in neurons exposed to NU-757 cells potentially explains the mechanistic underpinnings of the observed differences in neuronal morphology.

From a therapeutic perspective, our data demonstrate that pharmacological MEK inhibition significantly improves dendritic spine morphology and synaptic protein expression in U-87MG-exposed neurons. While MEK inhibition alone attenuated tumor cell spreading and invasion, it did not induce tumor cell death. However, by integrating proteomic findings from the neuronal compartment, we identified GB-induced suppression of neuronal PP2A components as a potential tumor-promoting mechanism. Co-treatment with a PP2A activator (DT-061) and MEK inhibitor not only strongly suppressed U-87MG expansion but also induced significant tumor cell death. This combinatorial approach highlights a rational strategy to co-target kinase and phosphatase imbalances that drive tumor aggressiveness and neuronal dysfunction.

Importantly, NU-757 cells were resistant to MEK inhibition, showing no impairment in growth or invasion. Again, this difference likely reflects intrinsic signaling diversity among GBM. While U-87MG appears to be dependent on MAPK signaling, NU-757 may rely on alternative oncogenic drivers that are not targeted by MEK inhibitors. These findings emphasize the need for personalized therapeutic strategies and caution against generalizing results from cell lines to patient-derived models. Our system provides a robust platform for identifying such differential dependencies and testing tailored interventions.

In summary, this study introduces a versatile, compartmentalized in vitro model that enables mechanistic and therapeutic exploration of GB-neuron interactions at high spatial and molecular resolution. Using this platform, we demonstrate that the tumor source shapes neuronal vulnerability, highlight MEK/ERK signaling and PP2A regulation as important signaling components involved in these responses, and validate a dual-compartment therapeutic approach that restores neuronal structure while suppressing GB growth. This human cell–based experimental system provides a tractable platform for preclinical evaluation of neuroprotective and anti-tumor therapeutics. Collectively, our work advances understanding of neuron-GB crosstalk and offers a useful experimental platform for developing therapeutic strategies that address both tumor burden and neural integrity in GB.

Although simplified, the dual-interface culture configuration used here captures several cellular interactions relevant to GB pathology, particularly those occurring at the infiltrative tumor margin where tumor cells encounter neuronal circuits and resident immune cells. At the same time, the model does not fully reproduce the structural and cellular complexity of GB tissue, which includes additional stromal and vascular elements as well as extracellular matrix heterogeneity. Therefore, the platform is best interpreted as a reductionist system designed to dissect early tumor-neuron signaling events, which can be complemented in future studies by more complex ex vivo models that retain native tumor architecture.

A limitation of this study is the lack of simultaneous secretome analysis from neurons and GB cells, which prevents a precise mechanistic description of the soluble signaling components that drive their functional connections. Furthermore, we used a specific neuronal subtype, limiting the generalizability. GB tumors form in various brain regions innervated by different neuronal populations, suggesting that the observed relationships are most likely dependent on specific local microenvironments. Therefore, future studies should investigate reciprocal secretome profiles to identify the molecular mediators that underpin neuron-GB communication and systematically evaluate how different neuronal subpopulations, particularly cholinergic neurons, given their crucial neuromodulatory role influence these events. Moreover, tumor chunk cultures that maintain the native cytoarchitecture and tumor microenvironment (TME) of patient-derived specimens represent a robust ex vivo platform for delineating neuron-specific interactions and responses within individual patient tumors.

## Supplementary Information

Below is the link to the electronic supplementary material.

Supplementary Material 1

Supplementary Material 2

Supplementary Material 3

Supplementary Material 4

## Data Availability

No datasets were generated or analysed during the current study.

## References

[1] Ostrom QT et al. (2020) CBTRUS statistical report: primary brain and other central nervous system tumors diagnosed in the United States in 2013–2017. Neurooncology 22:1–42 10.1093/neuonc/noaa200 PMC7596247 33123732

[2] Ostrom QT et al. (2021) CBTRUS statistical report: primary brain and other central nervous system tumors diagnosed in the United States in 2014–2018. Neurooncology 23:1–105 10.1093/neuonc/noab200 PMC8491279 34608945

[3] Faisal SM et al. (2025) Current landscape of preclinical models for pediatric gliomas: clinical implications and future directions. Cancers 17(13):2221 40647519 10.3390/cancers17132221 PMC12248539

[4] Rizwani F, Patil P, Jain K (2025) Unlocking glioblastoma: breakthroughs in molecular mechanisms and next-generation therapies. Med Oncol 42(7):276 40542948 10.1007/s12032-025-02830-1 PMC12182518

[5] Guo X et al. (2024) Neuronal activity promotes glioma progression by inducing proneural-to-mesenchymal transition in glioma stem cells. Cancer Res 84(3):372–387 37963207 10.1158/0008-5472.CAN-23-0609

[6] Johung T, Monje M (2017) Neuronal activity in the glioma microenvironment. Curr Opin Neurobiol 47:156–161 29096244 10.1016/j.conb.2017.10.009 PMC5927594

[7] Gillespie S, Monje M (2018) An active role for neurons in glioma progression: making sense of Scherer’s structures. Neuro Oncol 20(10):1292–1299 29788372 10.1093/neuonc/noy083 PMC6120364

[8] De Silva MI, Stringer BW, Bardy C (2023) Neuronal and tumourigenic boundaries of glioblastoma plasticity. Trends Cancer 9(3):223–236 36460606 10.1016/j.trecan.2022.10.010

[9] Krishna S et al. (2023) Glioblastoma remodelling of human neural circuits decreases survival. Nature 617(7961):599–607 37138086 10.1038/s41586-023-06036-1 PMC10191851

[10] Montgomery MK et al. (2020) Glioma-induced alterations in neuronal activity and neurovascular coupling during disease progression. Cell Rep 31(2):107500 32294436 10.1016/j.celrep.2020.03.064 PMC7443283

[11] Pei Z et al. (2020) Pathway analysis of glutamate-mediated, calcium-related signaling in glioma progression. Biochem Pharmacol 176:113814 31954716 10.1016/j.bcp.2020.113814 PMC8403340

[12] Sontheimer H (2008) A role for glutamate in growth and invasion of primary brain tumors. J Neurochem 105(2):287–295 18284616 10.1111/j.1471-4159.2008.05301.x PMC2557065

[13] Venkatesh HS et al. (2019) Electrical and synaptic integration of glioma into neural circuits. Nature 573(7775):539–545 31534222 10.1038/s41586-019-1563-y PMC7038898

[14] Piao Y et al. (2012) Glioblastoma resistance to anti-VEGF therapy is associated with myeloid cell infiltration, stem cell accumulation, and a mesenchymal phenotype. Neuro Oncol 14(11):1379–1392 22965162 10.1093/neuonc/nos158 PMC3480262

[15] Wild-Bode C et al. (2001) Sublethal irradiation promotes migration and invasiveness of glioma cells: implications for radiotherapy of human glioblastoma. Cancer Res 61(6):2744–2750 11289157

[16] Sprugnoli G, Golby AJ, Santarnecchi E (2021) Newly discovered neuron-to-glioma communication: new noninvasive therapeutic opportunities on the horizon? Neurooncol Adv 3(1):vdab018 33738449 10.1093/noajnl/vdab018 PMC7954106

[17] Venkatesh HS et al. (2017) Targeting neuronal activity-regulated neuroligin-3 dependency in high-grade glioma. Nature 549(7673):533–537 28959975 10.1038/nature24014 PMC5891832

[18] Venkatesh HS (2023) Targeting electrochemical communication between neurons and cancer. Sci Transl Med 15(706):eadi5170 37494471 10.1126/scitranslmed.adi5170

[19] Smirnova L, Hartung T (2024) The promise and potential of brain organoids. Adv Healthc Mater 13(21):e2302745 38252094 10.1002/adhm.202302745

[20] Liu Y et al. (2023) Patient-derived xenograft models in cancer therapy: technologies and applications. Signal Transduct Target Ther 8(1):160 37045827 10.1038/s41392-023-01419-2 PMC10097874

[21] Yadav N, Purow BW (2024) Understanding current experimental models of glioblastoma-brain microenvironment interactions. J Neurooncol 166(2):213–229 38180686 10.1007/s11060-023-04536-8 PMC11056965

[22] Varn FS et al. (2022) Glioma progression is shaped by genetic evolution and microenvironment interactions. Cell 185(12):2184–2199e16 35649412 10.1016/j.cell.2022.04.038 PMC9189056

[23] Mancusi R, Monje M (2023) The neuroscience of cancer. Nature 618(7965):467–479 37316719 10.1038/s41586-023-05968-y PMC11146751

[24] Silverman DA et al. (2021) Cancer-associated neurogenesis and nerve-cancer cross-talk. Cancer Res 81(6):1431–1440 33334813 10.1158/0008-5472.CAN-20-2793 PMC7969424

[25] Fernandopulle MS et al. (2018) Transcription factor–mediated differentiation of human iPSCs into neurons. Curr protocols cell biology 79(1):e51 10.1002/cpcb.51 PMC6993937 29924488

[26] Le NT et al. (2024) Prion protein pathology in Ubiquilin 2 models of ALS. Neurobiol Dis 201:106674 39299489 10.1016/j.nbd.2024.106674 PMC11651290

[27] Kaech S, Banker G (2006) Culturing hippocampal neurons. Nat Protoc 1(5):2406–2415 17406484 10.1038/nprot.2006.356

[28] Seker-Polat F et al. (2022) Tumor cell infiltration into the brain in glioblastoma: from mechanisms to clinical perspectives. Cancers 14(2):443 35053605 10.3390/cancers14020443 PMC8773542

[29] Allen M et al. (2016) Origin of the U87MG glioma cell line: good news and bad news. Sci Transl Med 8(354):354re3 27582061 10.1126/scitranslmed.aaf6853

[30] Tallman MM et al. (2021) The small molecule drug CBL0137 increases the level of DNA damage and the efficacy of radiotherapy for glioblastoma. Cancer Lett 499:232–242 33253788 10.1016/j.canlet.2020.11.027 PMC7779703

[31] Corrêa SA, Eales KL (2012) The role of p38 MAPK and its substrates in neuronal plasticity and neurodegenerative disease. J Signal Transduct 2012:p649079 10.1155/2012/649079 PMC3389708 22792454

[32] Gorai S, Rathore G, Das K (2024) Selumetinib-a comprehensive review of the new FDA-approved drug for neurofibromatosis. Indian Dermatol Online J 15(4):701–705 39050082 10.4103/idoj.idoj_569_23 PMC11265740

[33] Kelemen BR, Hsiao K, Goueli SA (2002) Selective in vivo inhibition of mitogen-activated protein kinase activation using cell-permeable peptides. J Biol Chem 277(10):8741–8748 11756441 10.1074/jbc.M108459200

[34] Sangodkar J et al. (2017) Activation of tumor suppressor protein PP2A inhibits KRAS-driven tumor growth. J Clin Invest 127(6):2081–2090 28504649 10.1172/JCI89548 PMC5451217

[35] Venkataramani V et al. (2022) Glioblastoma hijacks neuronal mechanisms for brain invasion. Cell 185(16):2899–2917e31 35914528 10.1016/j.cell.2022.06.054

[36] Tetzlaff SK et al. (2025) Characterizing and targeting glioblastoma neuron-tumor networks with retrograde tracing. Cell 188(2):390–411e36 39644898 10.1016/j.cell.2024.11.002

[37] Zeng Y et al. (2018) The tumorgenicity of glioblastoma cell line U87MG decreased during serial in vitro passage. Cell Mol Neurobiol 38(6):1245–1252 29948550 10.1007/s10571-018-0592-7 PMC11481925

[38] Zeng X et al. (2017) The expression of G protein-coupled receptor kinase 5 and its interaction with dendritic marker microtubule-associated protein-2 after status epilepticus. Epilepsy Res 138:62–70 29080472 10.1016/j.eplepsyres.2017.10.011

[39] Venkataramani V et al. (2019) Glutamatergic synaptic input to glioma cells drives brain tumour progression. Nature 573(7775):532–538 31534219 10.1038/s41586-019-1564-x

[40] Zeng Q et al. (2019) Synaptic proximity enables NMDAR signalling to promote brain metastasis. Nature 573(7775):526–531 31534217 10.1038/s41586-019-1576-6 PMC6837873

[41] Le N et al (2024) Prion protein pathology in Ubiquilin 2 models of ALS. Neurobiol Dis 201:106674 10.1016/j.nbd.2024.106674 10.1016/j.nbd.2024.106674 PMC11651290 39299489

