# Supplementary material for: Dissecting acute neuronal responses to glioblastoma using a dual-interface human iPSC neuronal culture platform
Source: Acta Neuropathol Commun. 2026 May 16;14:149. doi: 10.1186/s40478-026-02312-z (PMC13371652; doi:10.1186/s40478-026-02312-z)

Fig. 2: Full-length gels/blots images

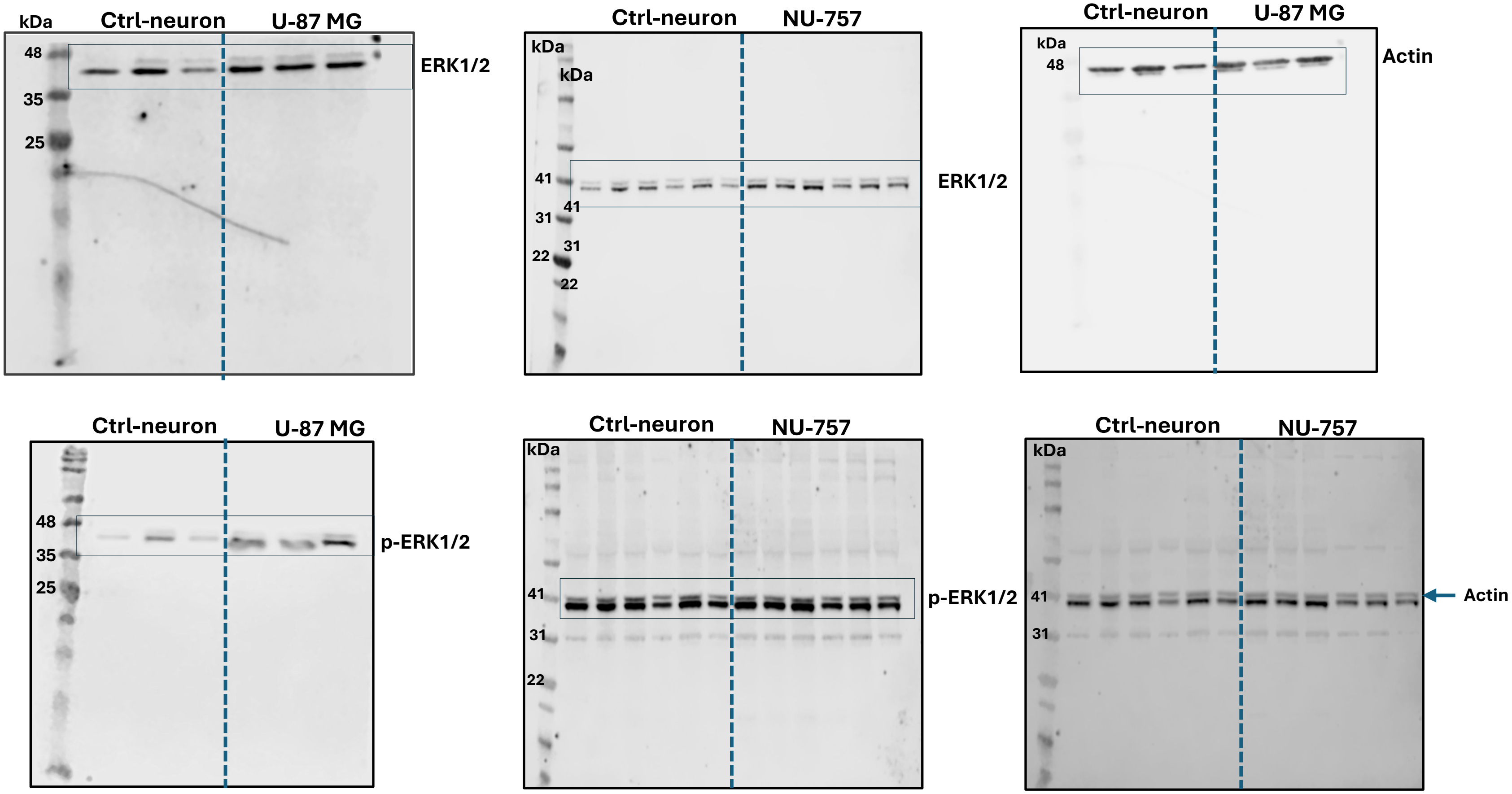

Fig. S1: Full-length gels/blots images

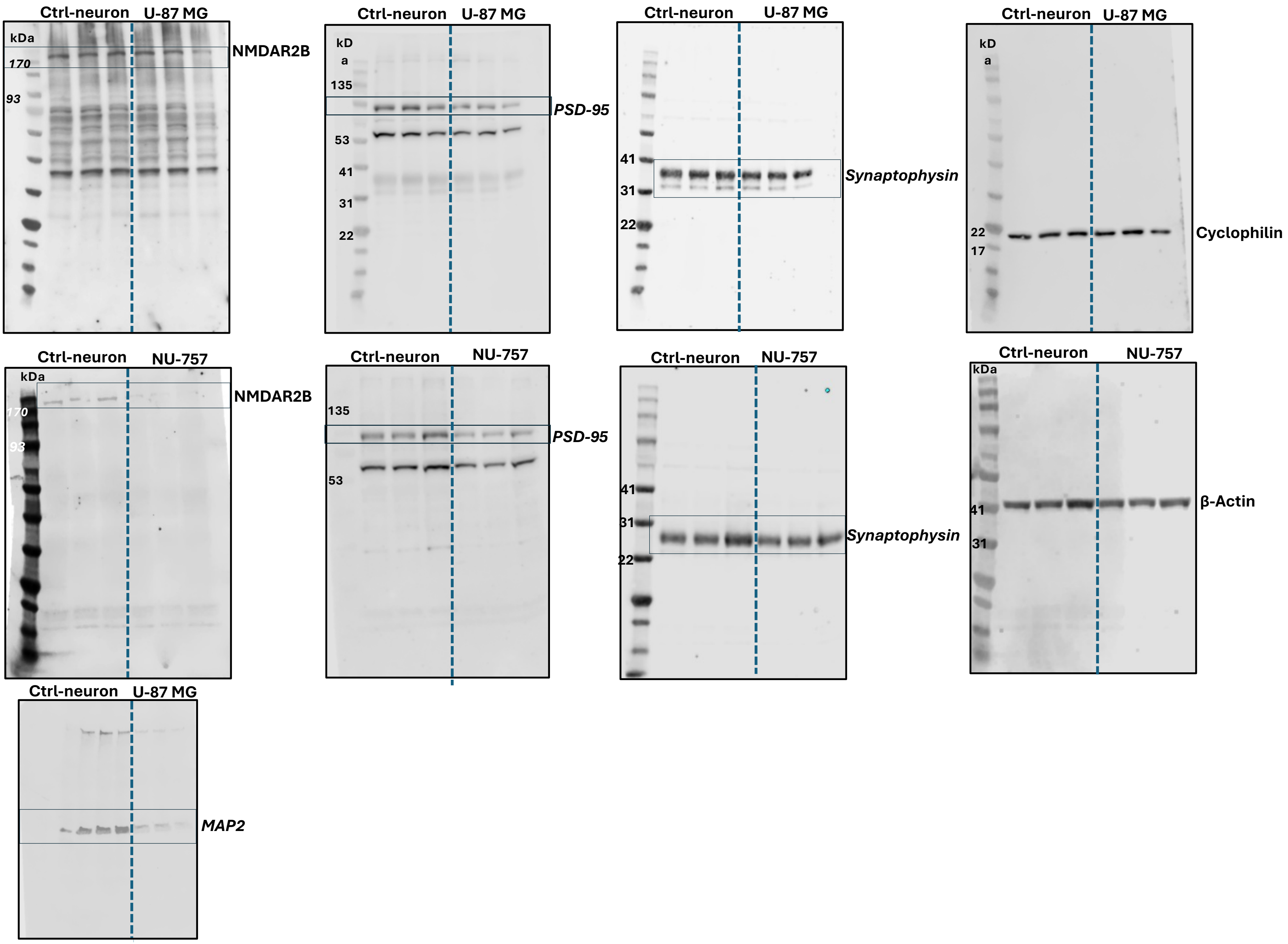

Fig S4: Full-length gels/blots images

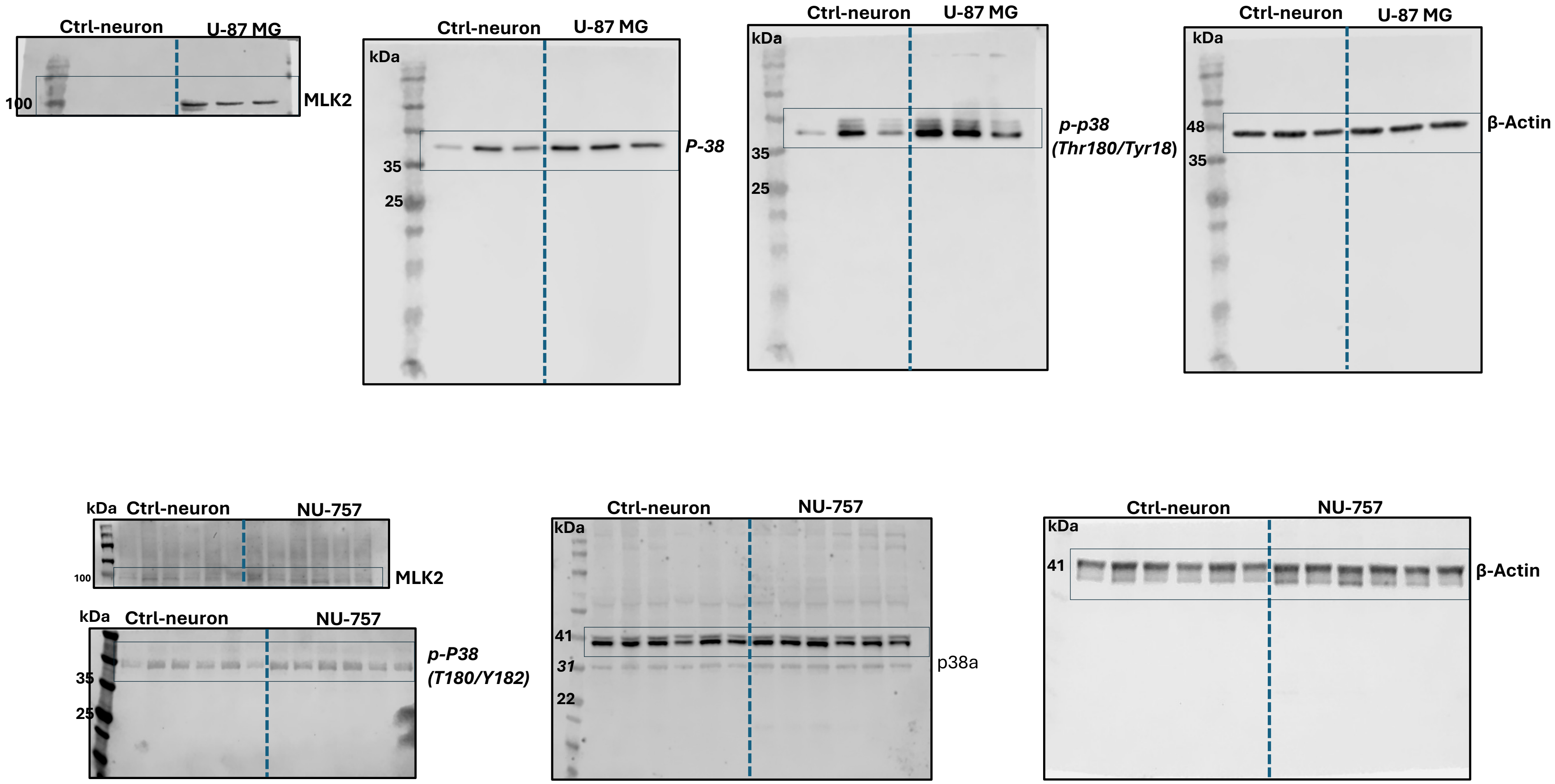

Fig S5: Full-length gels/blots images

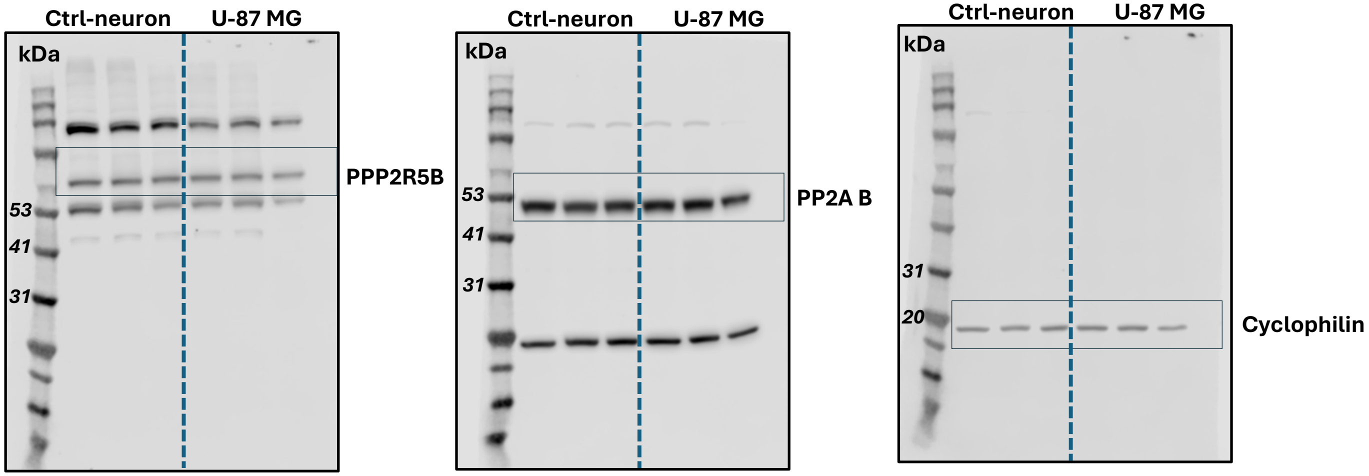

Supplement: Supplementary file 1 — Supplementary Material 1 [file 40478_2026_2312_MOESM1_ESM.pdf]
