# Supplementary material for: Dissecting acute neuronal responses to glioblastoma using a dual-interface human iPSC neuronal culture platform
Source: Acta Neuropathol Commun. 2026 May 16;14:149. doi: 10.1186/s40478-026-02312-z (PMC13371652; doi:10.1186/s40478-026-02312-z)
Supplement: Supplementary file 2 — Supplementary Material 2 [file 40478_2026_2312_MOESM2_ESM.pdf]

Dissecting Acute Neuronal Responses to Glioblastoma Using a Dual-Interface Human iPSC Neuronal Culture Platform

Ouada Nebie[1,2], Niyi Adelakun[1,2,3], Brian Fries [4], Luke Kollin[5], Liwen Zhang[4], Akhil Medikonda[1], Monica Venere[5], Pierre Giglio[6, 7], Nam Chu[1,2,3#] and Nhat Le[1#]

(1) *Department of Cancer Biology and Genetics, College of Medicine, The Ohio State University Wexner Medical Center, Columbus, OH 43210, USA*

(2) *Comprehensive Cancer Center, The Ohio State University Wexner Medical Center, Columbus, OH 43210, USA*

(3) *The Ohio State Biochemistry Program (OSBP), The Ohio State University, Columbus, OH 43210, USA*

(4) *Campus Chemical Instrument Center, Mass Spectrometry and Proteomics, The Ohio State University, Columbus, OH 43210, USA*

(5) *Department of Radiation Oncology, The Ohio State University Comprehensive Cancer Center, Columbus, OH 43210, USA.*

(6) *Department of Neurology, The Ohio State University Comprehensive Cancer Center, College of Medicine, The Ohio State University Wexner Medical Center, Columbus, OH 43210, USA*

(7) *James Cancer Hospital and Solove Research Institute Comprehensive Cancer Center, The Ohio State University Comprehensive Cancer Center, College of Medicine, The Ohio State University Wexner Medical Center, Columbus, OH 43210, USA*

[#] *Corresponding authors: nhat.le@osumc.edu; nam.chu@osumc.edu*

MATERIALS AND METHODS

Cell line cultures

The serum adapted U-87 MG (ATCC HTB-14) and HMC3 (ATCC CRL-3304) cell lines were maintained in Dulbecco's Modified Eagle Medium (DMEM; Gibco, Cat. #1196511) supplemented with 10% fetal bovine serum (FBS; Gibco, Cat. #1600044), 100 U/mL penicillin, and 100 µg/mL streptomycin (Gibco, Cat. #15070063). Cultures were incubated at 37 °C in a humidified atmosphere containing 5% CO_2.

The serum free patient-derived glioblastoma cell line NU-757 originated from primary human brain tumor tissue, obtained as de-identified specimens from the Northwestern University Nervous System Tumor Bank [1, 2]. NU-757 cells were cultured under adherent, serum-free conditions in phenol red–free Neurobasal medium (Gibco, Cat. #12348017) supplemented with B-27 Supplement minus Vitamin A (Gibco, Cat. #12587010), human FGF-2 (10 ng/mL; Miltenyi Biotec, Cat. #130-097-751), human EGF (10 ng/mL; Miltenyi Biotec, Cat. #130-093-842), GlutaMAX (1×; Gibco, Cat. #35050061), sodium pyruvate (1 mM; Gibco, Cat. #11360070), and penicillin/streptomycin (100 U/mL and 100 µg/mL, respectively; Gibco, Cat. #15070063). NU-757 cells were seeded onto tissue culture plates pre-coated with Geltrex LDEV-free stem cell, a qualified reduced-growth factor basement membrane matrix (Life Technologies, Cat. #A1413302). Coating was performed by incubating plates with Geltrex for 4 hours at 37 °C or overnight at 4 °C. For passaging, NU-757 cells were dissociated into single-cell suspensions using TrypLE Express (phenol red–free; Gibco, Cat. #12604013).

Human iPSCs and iPSC-derived cortical neuron culture

Human induced pluripotent stem cells (iPSCs) of the KOLF2.1 line (Jackson Laboratory for Genomic Medicine) were cultured in mTeSR™ Plus medium (STEMCELL Technologies) on Matrigel-coated 6-well plates (BD Matrigel™ hESC-qualified Matrix, Cat. #354277; coating time for 30 minutes at room temperature). Routine passaging was performed using ReLeSR™ (STEMCELL Technologies), an enzyme-free dissociation reagent, and medium was replaced daily to support optimal cell growth.

Differentiation of iPSCs into cortical neurons was carried out as previously described [3], using an established neurogenin-2 (NGN2)-induction protocol [4, 5]. Briefly, human iPSCs stably carrying the NGN2 transgene were dissociated into single cells and plated on Matrigel-coated plates in Induction Medium (IM) supplemented with doxycycline (2 μg/mL) to induce NGN2 expression and the ROCK inhibitor Y-27632 (10 μM; Tocris Bioscience, Cat. #1254) to promote cell survival. Cells were maintained in induction conditions for 3 days. On Day 4 of differentiation, cells were detached using Accutase (Gibco, Cat. #A1110501) and replated at defined densities onto poly-D-lysine (PDL; Gibco, Cat. #A3890401) and poly-L-ornithine (PLO; Sigma, Cat. #P4957-50mL)- coated 12-well plates. For coating, each well received 700 μL of PDL solution, which was incubated for 4 hours at room temperature. PDL was then removed and replaced with an equal volume of PLO solution, which was incubated overnight at 4 °C. Before cell seeding, the wells were rinsed three times with sterile PBS (5 minutes per wash).

Following replating, cells were cultured in Neuronal Culture Medium (CM). Medium was partially replaced (50%) daily using pre-warmed CM. Neurons were maintained for at least 35 days prior to experimental use. Neurons were differentiated for 35 days prior to experimentation, a stage at which this differentiation protocol has been previously

shown to generate mature neurons with established dendritic arborization neuronal and synaptic marker expression [3, 4].

Dual-interface co-culture system

This interface human neuronal culture system is adapted from previously described methods for culturing rodent embryonic hippocampal neurons on coverslips suspended above a glial monolayer [6-8]. Briefly, glass coverslips were first sterilized by autoclaving. Once dry, three wax legs were applied to each coverslip to act as spacers. To create the legs, a Pasteur pipette was dipped into molten paraffin and used to place three evenly spaced dots near the periphery of each coverslip. The wax legs were approximately 0.5 mm in height and 1.0-2.0 mm in diameter, providing physical separation between the neuronal and glial layers during co-culture.

After the wax solidified, each coverslip was transferred to a well of a 12-well culture plate and sterilized under UV light for one hour. Coverslips were then coated sequentially with poly-D-lysine and poly-L-ornithine as described above to promote cell adhesion. Human induced pluripotent stem cells (iPSCs) were subsequently seeded and differentiated directly on the coated coverslips into mature cortical neurons as described above.

For co-culture experiments, differentiated neurons on coverslips were suspended above a monolayer of human microglial HMC3 cells seeded with either U-87 MG or serum free patient-derived NU-757 glioblastoma cells. Neurons were maintained in this dual-interface configuration for 24 hours prior to downstream analyses.

Neurons and glioblastoma co-culture in the dual-interface culture system

In this system, GB and microglial cells were first co-seeded onto 12-well plates one day prior to the addition of neurons (Day 1). Cells were plated at a 1:1 ratio, while control wells contained only HMC3 cells. Plates were incubated overnight at 37 °C with 5% CO_2. On the day of co-culture initiation (Day 0), the medium in the GB/microglia cultures was replaced with fresh neuronal culture medium (CM). Coverslips with differentiated neurons were then carefully lifted from their original plates and inverted onto the top of the GB/HMC3 or control HMC3 cell layers. This orientation positioned the neuronal layer facing the GB/microglia layer while maintaining a defined physical separation of approximately 0.5 mm, created by the wax feet on the coverslips.

This dual-interface setup enabled exchange of diffusible factors between neurons and GB/microglia cells without allowing direct cell-cell contact. Co-cultures were incubated for 24 hours before neurons were collected for downstream analyses, including proteomics, western blotting, or immunostaining.

Western blot analysis

Cells were collected and lysed in 1× cell lysis buffer (Cell Signaling Technology, Cat. #9803) supplemented with a protease and phosphatase inhibitor cocktail (Thermo Scientific, Cat. #A32959). Total protein concentrations were determined using the bicinchoninic acid (BCA) assay (Pierce, Cat. #23225), and samples were stored at -80 °C until further use.

For electrophoresis, 10-25 μg of total protein per sample were resolved on 4-20% gradient Bis-Tris precast gels (GenScript, NJ, USA) and transferred to 0.45 μm nitrocellulose membranes (Bio-Rad, CA, USA). Membranes were blocked with 5% bovine serum albumin (BSA; Gold Biotechnology, Cat. #A-420-500) in TBS buffer for 1 hour at

room temperature, then incubated overnight at 4 °C with the primary antibodies (listed in Table S1). Following primary antibody incubation, membranes were washed and incubated with secondary antibodies (listed in Table S1). Protein bands were visualized using the LI-COR Odyssey Imaging System. Densitometric quantification of Western blot bands was carried out using ImageJ software.

Immunocytochemistry

Cells grown on glass coverslips were washed with phosphate-buffered saline (PBS, 1x) and fixed in 4% paraformaldehyde for 12 minutes at room temperature. For detection of intracellular antigens, cells were permeabilized with 0.1% Triton X-100 in PBS for 5 minutes prior to blocking. Non-specific binding was blocked with 1% bovine serum albumin (BSA) in PBS for 30 minutes at room temperature (RT).

Primary antibodies (listed in Table S1) were diluted in 1% BSA/PBS and applied to cells for 2 hours at RT. After washing with PBS, fluorophore-conjugated secondary antibodies diluted in 1% BSA/PBS were applied for 1 hour at room temperature in the dark. Cells were then washed five times (5 minutes each) in PBS and counterstained with DAPI (Invitrogen, Cat. #D1306) to visualize nuclei. Coverslips were mounted using Vectashield Mounting Medium (Vector Laboratories, Cat. #H-1900) and stored at 4°C until imaging.

All imaging was performed using Olympus FV3000 Multi-Photon Confocal Microscopes with identical acquisition settings across conditions. A 63x oil immersion objective (numerical aperture, NA=1.4) was used for high-resolution image capture.

Dendritic Spine Analysis

Dendritic spine density was assessed to evaluate synaptic effects, as previously described [3, 7]. Neurons were exposed in different culture conditions, followed by fixation in 4% paraformaldehyde. Neurons cultured on coverslips were then stained with either Alexa Fluor 488-phalloidin or rhodamine-phalloidin to label F-actin-rich dendritic spines. Confocal images were acquired using Olympus FV3000 Multi-Photon Confocal Microscopes equipped with 63x oil and 100x oil immersion objectives (NA = 1.4 numerical aperture, NA=1.4).

For quantification, 5-10 isolated dendritic segments were selected per image. Images were processed in ImageJ (FIJI) using a threshold optimized to include dendritic spine outlines while excluding background fluorescence [9]. Spine numbers were normalized to the corresponding dendritic length and reported as spines per micrometer (spines/μm). For each condition, 5-10 images were analyzed across 2-3 independent cultures.

Immunofluorescence Intensity Quantification

Fluorescence intensity within defined regions of interest (ROIs) was quantified using ImageJ (FIJI). For each sample, ROIs were manually selected based on the area of interest, such as dendritic spines, dendrites, or intracellular compartments. Each image was processed to ensure uniformity, including background subtraction and optimization of thresholding to exclude non-specific fluorescence signals. The mean fluorescence intensity per pixel was calculated by dividing the integrated density by the ROI area. To control for variability between experimental conditions, all fluorescence intensities were normalized to the average intensity of the control group. Data from at least 5 randomly

selected fields per condition were quantified to ensure robust sampling. For each condition, 15-24 neurons from 2-3 independent cultures were analyzed.

Proteomics analysis

Sample preparation and protein digestion via STrap

For each proteomics experiment, neuron control samples (neurons cultured in the dual-interface system without glioblastoma cells) were prepared using the same neuronal differentiation batch as those exposed to GB cells (U-87 MG or NU-757) to avoid batch effects. Each culture condition included four biological replicates. Neuron cultures were harvested in a lysis buffer containing 5% SDS in 50 mM triethylammonium bicarbonate (TEAB; Sigma, Cat. #T7408) for protein extraction. Samples were centrifuged at 10,000 × g for 10 minutes at 4 °C, and the resulting supernatant was collected for subsequent proteomic analysis. Total protein concentrations were quantified using a bicinchoninic acid (BCA) protein assay kit (Pierce) according to the manufacturer's instructions.

Samples were then prepared for trypsin digestion using the S-Trap method. Five microliters of 50 mM ABC containing 5 µg/µL DTT were added to each sample, followed by incubation at 65 °C for 15 minutes. Next, 5 µL of 50 mM ABC containing 15 µg/µL iodoacetamide were added and incubated at room temperature for 15 minutes in the dark. Samples were acidified by adding 12% phosphoric acid (1:10 v/v acid to sample). For every 25 µL of sample, 165 µL of TEAB (1 M)/methanol (10:90 v/v) was added and loaded onto the S-Trap column for further processing.

Samples were centrifuged at 4,000 × g for 3 minutes at 4 °C to remove the supernatant. The column was washed 3-6 times with 150 µL of TEAB/methanol (10:90 v/v), depending on the initial loading volume. After the final wash, sequencing-grade

trypsin dissolved in 50 mM TEAB was added, and digestion was carried out overnight at 37 °C.

The next day, peptides were sequentially eluted with 40 µL of 50 mM TEAB, 0.1% formic acid (FA), and 0.1% FA in 50% acetonitrile. The eluates were pooled, dried using a vacuum concentrator, and resuspended in 20 µL of 50 mM acetic acid. Peptide concentration was determined by absorbance at 280 nm using a Nanodrop spectrophotometer.

LC-MS/MS on Eclipse

Nano-liquid chromatography-nanospray tandem mass spectrometry (Nano-LC/MS/MS) for protein identification was performed using a Thermo Scientific Orbitrap Eclipse mass spectrometer. Samples (1 µg) were separated on an EASY-Spray nano column (PepMap™ RSLC, C18, 3 µm, 100 Å, 75 µm × 250 mm; Thermo Scientific) using a 2D RSLC HPLC system (Thermo Scientific). Each sample was injected into a µ-Precolumn Cartridge (Thermo Scientific) and desalted with 0.1% formic acid in water for 5 minutes. The injector port was then switched to inject mode, and peptides were eluted from the trap onto the analytical column. Mobile phase A consisted of 0.1% formic acid in water, and mobile phase B was acetonitrile with 0.1% formic acid. The flow rate was set at 300 nL/min. Mobile phase B was increased from 2% to 16% over 105 minutes, then from 16% to 25% over 10 minutes, followed by an increase from 25% to 85% over 1 minute. The column was held at 95% B for 4 minutes before being returned to 2% B in 1 minute. The column was equilibrated at 2% B (98% A) for 15 minutes before the next sample injection.

MS/MS data were acquired with a spray voltage of 1.95 kV and a capillary temperature of 305 °C. The scan sequence was based on the preview mode data-dependent TopSpeed™ method. Full MS scans were recorded between m/z 375-1500, followed by MS/MS scans of the most abundant peaks in the next 3 × 1 seconds. Full scans were acquired in Fourier Transform (FT) mode at a resolution of 120,000 with internal mass calibration for high mass accuracy. Three compensation voltages (CV = -40, -60, and -80 V) were applied for acquisition. The AGC target for FT full scans was set to 4×10^5 ions, with the maximum injection time set to “Auto” and one microscan. MS^n was performed using higher-energy collisional dissociation (HCD) in Orbitrap mode to ensure accurate mass detection of post-translational modifications. The HCD collision energy was set at 30%. The AGC target for ion trap MS^n scans was 1×10^4 ions, with an “Auto” maximum injection time and one microscan. Dynamic exclusion was enabled with a repeat count of 1 within 20 seconds and a low and high mass width of ±10 ppm.

Database search

The resulting MGF files generated from the samples were searched using Mascot Daemon (Matrix Science, version 2.7.0, Boston, MA) against both the RSSB sequence and the human protein database. The precursor ion mass accuracy was set to 10 ppm, and the search parameters included allowance for the accidental selection of one ^13C peak. Fragment mass tolerance was set to 0.5 Da. Variable modifications considered during the search included acetylation (K), phosphorylation (S and T), oxidation (M), deamidation (N and Q), and carbamidomethylation (C). Up to four missed cleavages by the enzyme were permitted. A decoy database was also searched to determine the false discovery rate (FDR), and peptides were filtered accordingly. The significance threshold

was set at $p < 0.05$, and valid peptide identifications required bold red peptide matches. Proteins were considered valid if they had an FDR of less than 1% and a minimum of two significant peptides. All modified peptides were manually validated.

Quantitation and bioinformatics

Relative quantitation was performed using a label-free quantitation approach based on mass spectral peak intensities. Peptide precursor (MS1) intensities from both modified and unmodified peptides were extracted from the Proteome Discoverer MASCOT search results and summed for quantitative comparison. Protein intensities were normalized to total protein intensity, and “low abundance resampling” was applied as the imputation mode. Statistical significance of expression differences between groups was evaluated using a student’s t-test. Proteins with a p-value < 0.05 and a fold change ≥ 2 were considered upregulated, while those with a fold change ≤ 0.5 were considered downregulated.

The data underwent bioinformatics analyses using publicly available online databases including, Metascape [10] and Cytoscape [11]. For comparative pathway and network enrichment analysis, protein list enrichment and network analyses were performed using the Metascape platform [10]. Briefly, for each given gene set encoded from different expressed protein list, enrichment analyses have been carried out. Two defined gene sets ("NU-757" and "U-87 MG") were analyzed independently and in combination. Gene identifiers were mapped to *Homo sapiens* Entrez Gene IDs, and redundancy was resolved prior to analysis. Functional enrichment analyses were conducted across multiple ontology sources including GO Biological Processes, GO Molecular Functions, GO Cellular Components, Reactome, KEGG, Hallmark, and

BioCarta. Enriched terms were identified using a cumulative hypergeometric distribution with a cutoff of $p < 0.01$, enrichment factor > 1.5, and a minimum gene count of 3. Multiple testing correction was applied using the Benjamini-Hochberg procedure to calculate q-values. To reduce redundancy and visualize the biological landscape, enriched terms were hierarchically clustered using Kappa-statistic-based similarity (>0.3), with the most statistically significant term within each cluster serving as its representative. Up to 20 top clusters were visualized via heatmaps and enrichment networks. Protein-protein interaction (PPI) networks were built using data from STRING (physical score > 0.132), BioGRID, OmniPath, and InWeb_IM. The MCODE algorithm was applied to detect densely connected network modules, and enrichment analysis was performed on each module individually to annotate biological functions. Further characterization was conducted using curated gene set libraries including DisGeNET. All enrichment results were filtered based on standard thresholds ($p < 0.01$, enrichment factor > 1.5) and summarized by most significant terms per cluster. Data visualizations, including network maps (rendered with Cytoscape), were generated to explore shared and unique pathway associations across gene lists.

Glioblastoma Cell Migration and Invasion Assay

To assess the migratory and invasive properties of glioblastoma (GB) cells, NU-757 and U-87 MG cells were seeded at low density in Boyden chambers with or without Geltrex-coated membranes. For this experiment, Millicell 12-well hanging cell culture inserts (Merck Millipore, cat. PTEP12H48) and HTS Transwell-96 permeable supports (Corning, cat. #3374) with 8.0 μm pore polyester membrane were utilized. The pore size

was selected based on the migratory cells being used and the inserts are appropriate for evaluating cell migration and invasion.

For the cell migration/invasion assay, inserts were placed into receiver plates. Pre-warmed cell culture medium without cells was added to the basolateral compartment of each well. Next, cell suspension was prepared and seeded onto the apical side of the inserts: 100 µL per well for 96-well inserts (HTS Transwell-96) and 700 µL per insert for 12-well inserts (Millicell). An identical cell number was seeded across all conditions. Plates were then incubated at 37°C in a humidified CO_2 incubator. Finally, the migrated/invaded cells accumulating in the receiver wells were monitored over time using the IncuCyte system (IncuCyte S3, Sartorius). To validate the assay system and minimize bias, each condition was performed in at least triplicate. Furthermore, a non-invasive cell line, the HMC3 cells, was included as a control to confirm the barrier membrane effectively inhibited invasion.

In inhibition experiments, cells were seed as above and treated twenty-four hours after with varying concentrations of a MEK inhibitor (selumetinib, MedChemExpress, Cat. # HY-50706), ERK inhibitor peptide [12], or a PP2A activator (DT-061, MedChemExpress, Cat. # HY-112929). Plates were placed into the IncuCyte system (IncuCyte S3, Sartorius), housed within a normal tissue culture incubator (37°C, 5% CO_2). The Incucyte® Live-Cell Analysis Systems scan the tissue culture plates at predetermined intervals to monitor cultures and generate quantified kinetic data. Here 5 or 49 images per well were acquired using a 10x objective every six hours over a period of five to seven days. This live-cell imaging allowed for continuous monitoring of migrated cells and cell proliferation and the generation of growth and growth inhibition curves via the basic analyzer function and the

phase image sharpness metric, which is automatically calculated by the IncuCyte software. As cells proliferate, they generate additional edges and density, increasing contrast and sharpness. For the analysis, we chose the desired time periods, experimental settings, number of images and the replicates, then, a masking process was initiated to eliminate the background so that the cells underneath could be seen clearly. To have a consistent comparison between the conditions, the starting point was also normalized. Finally, the graph and the raw data generated by the software were exported and used accordingly

Statistical Analysis

All statistical analyses were conducted using GraphPad Prism version 10.0.0. Data are presented as mean ± standard error of the mean (SEM). Comparisons between two groups were performed using unpaired two-tailed t-tests, while comparisons across multiple groups were assessed using one-way ANOVA followed by Tukey's post-hoc test for multiple comparisons. Details regarding the number of replicates (n), type of statistical test used, and significance levels are provided in the corresponding figure legends.

REFERENCES

1. Tallman, M., et al., *The small molecule drug CBL0137 increases the level of DNA damage and the efficacy of radiotherapy for glioblastoma.* Cancer Letters, 2020. **499**.
2. Tallman, M.M., et al., *Improving Localized Radiotherapy for Glioblastoma via Small Molecule Inhibition of KIF11.* Cancers (Basel), 2023. **15**(12).
3. Le, N.T., et al., *Prion protein pathology in Ubiquilin 2 models of ALS.* Neurobiol Dis, 2024. **201**: p. 106674.
4. Fernandopulle, M.S., et al., *Transcription factor–mediated differentiation of human iPSCs into neurons.* Current protocols in cell biology, 2018. **79**(1): p. e51.
5. Wang, C., et al., *Scalable production of iPSC-derived human neurons to identify tau-lowering compounds by high-content screening.* Stem cell reports, 2017. **9**(4): p. 1221-1233.
6. Kaech, S. and G. Banker, *Culturing hippocampal neurons.* Nat Protoc, 2006. **1**(5): p. 2406-15.

7. Fang, C., et al., *Prions activate a p38 MAPK synaptotoxic signaling pathway.* PLoS Pathog, 2018. **14**(9): p. e1007283.
8. Le, N.T.T., B. Wu, and D.A. Harris, *Prion neurotoxicity.* Brain Pathol, 2019. **29**(2): p. 263-277.
9. Srivastava, D.P., K.M. Woolfrey, and P. Penzes, *Analysis of dendritic spine morphology in cultured CNS neurons.* J Vis Exp, 2011(53): p. e2794.
10. Zhou, Y., et al., *Metascape provides a biologist-oriented resource for the analysis of systems-level datasets.* Nat Commun, 2019. **10**(1): p. 1523.
11. Shannon, P., et al., *Cytoscape: a software environment for integrated models of biomolecular interaction networks.* Genome Res, 2003. **13**(11): p. 2498-504.
12. Kelemen, B.R., K. Hsiao, and S.A. Goueli, *Selective in vivo inhibition of mitogen-activated protein kinase activation using cell-permeable peptides.* J Biol Chem, 2002. **277**(10): p. 8741-8.

SUPPLEMENTAL DATA

Figure S1. Synaptic abnormalities in neurons in response to glioblastoma-derived signals.

Neurons from dual-interface co-cultures were analyzed for synaptic protein expression across three groups: control neurons (Ctrl-neuron), neurons exposed to U-87MG (U-87MG-neuron), and neurons exposed to NU-757 (NU-757-neuron).

(A-B) Western blot analysis was performed to assess the expression of synaptophysin (presynaptic marker), PSD-95 and NMDAR2B (postsynaptic markers), with actin used as a loading control. Representative immunoblots (A) and corresponding densitometric quantification (B) revealed a significant reduction in PSD-95 and NMDAR2B expression in neurons exposed to U-87MG and NU-757, indicating impaired postsynaptic signaling. Data are presented as mean ± SEM. Significance: ns (not significant), $^{**}P < 0.01$, $^{***}P < 0.001$, $^{****}P < 0.0001$, exposed neurons vs. control neurons, using unpaired t-tests.

(C–D) Neuronal MAP-2 (Microtubule-Associated Protein 2) expression was assessed in control and U-87 MG-exposed neurons. Representative immunofluorescence images (C) showed markedly reduced MAP-2 signal (green) in glioblastoma-exposed neurons, consistent with western blot results (D). Data represent 18-21 neuron segments. Values are shown as mean ± SD. Unpaired t-tests were used for statistical analysis; $^{*}P < 0.05$. (D) Western blots were probed for MAP-2, with actin as a loading control. Quantitative densitometric analysis confirmed a significant decrease in MAP-2 expression in glioblastoma-exposed neurons. Data represent three independent experiments ($n = 3$) and are presented as mean ± SEM. $^{**}P < 0.01$, exposed neurons vs. control, using unpaired t-tests.

Figure S2. Quantitative proteomics highlights differentially regulated proteins in neurons exposed to glioblastoma.

Quantitative proteomic analysis was performed on neurons exposed to conditioned media from U-87MG (Neurons_U-87), NU-757 (Neurons_NU-757), and untreated control neurons (n = 4 per group).

(A) Venn diagram showing the total number of proteins detected in Neurons_U-87 and Neurons_NU-757. More than 5,000 proteins were identified in each group, with 4,965 proteins shared, indicating a highly overlapping baseline proteome.

(B) Differential expression analysis using a fold change threshold of >1.5 or <0.5 revealed 91 and 520 differentially expressed proteins in Neurons_U-87 and Neurons_NU-757, respectively. Only 8 proteins were commonly dysregulated, suggesting distinct proteomic responses to each glioblastoma line.

(C) Venn diagram of significantly dysregulated proteins ($p < 0.05$) showing 36 proteins commonly altered in both Neurons_U-87 and Neurons_NU-757, indicating a partially shared but predominantly cell type-specific neuronal response to glioblastoma-derived factors.

Figure S3. Distinct proteomic profiles and functional enrichments in neurons exposed to patient-derived versus standard glioblastoma cells.

(A) Network visualization of enriched functional terms from differentially expressed proteins in neurons exposed to NU-757 versus U-87 MG glioblastoma cells for 24 hours.

Nodes are colored by cluster ID, with nodes sharing the same ID positioned near each other to indicate functional similarity.

(B) The same enrichment network as in (A), represented using pie charts. Each node is color-coded by the contributing protein list: NU-757 (red) or U-87 MG (blue). **(C-D)** Volcano plots of differentially expressed proteins in neurons exposed to U-87 MG (C) and NU-757 (D) glioblastoma cells compared to control neurons. Proteins are plotted by statistical significance ($-\log_{10}P$ value, y-axis) and fold change ($\log_2$ FC, x-axis). Significance thresholds are set at fold change (FC) ≥ 1.3 and P < 0.05. Red dots indicate significantly upregulated proteins, blue dots indicate significantly downregulated proteins, and grey dots indicate proteins with no significant change.

Figure S4. Alteration of MLK2 and p38 MAPK signaling pathways in neurons exposed to glioblastoma cells.

(A-B) Immunoblot analysis of neuronal lysates from dual-interface co-cultures, including control neurons (Ctrl-neuron), neurons exposed to U-87 MG glioblastoma cells (U-87 MG-neuron), and neurons exposed to patient-derived NU-757 glioblastoma cells (NU-757-neuron). Blots were probed for MLK2, total p38, phosphorylated p38 (p-p38, Thr180/Tyr182), and actin as a loading control. Data represent three independent experiments (n = 3). Lower panel (B) showed the densitometric quantification of western blot images. Values are presented as mean ± SEM. Statistical comparisons were performed using unpaired t-tests. Significance is indicated as: ns (not significant), *P < 0.05, ****P < 0.0001.

(C) Representative triple immunofluorescence images showing phalloidin (gray), p38 (green), and MLK2 (red) in neurons following 24-hour exposure to glioblastoma. DAPI (blue) marks nuclei. Dotted lines delineate nuclear and dendritic spine regions based on phalloidin and DAPI staining. Scale bars = 10 μm (nuclear panels) and 2 μm (spine panels).

(D-E) Quantification of p38 (D) and MLK2 (E) immunofluorescence intensity in neuronal nuclei and dendritic spines from (C). Data were pooled from 62-82 nuclear regions and 997-2163 spine regions across three independent experiments. Values are presented as mean ± SEM. Statistical comparisons were performed using unpaired t-tests. Significance is indicated as: ns (not significant), ****$P < 0.0001$.

Figure S5. PP2A involvement in ERK signaling and differential drug responses in glioblastoma models.

(A) Protein–protein interaction (PPI) network centered on ERK1/2 (MAPK3) and PP2A subunits (PPP2R1B and PPP2R5B), showing significantly altered proteins in neurons exposed to U-87 MG glioblastoma.

(B) Immunoblot analysis of neuronal lysates from dual-interface co-cultures, including control neurons (Ctrl-neuron) and neurons exposed to U-87 MG glioblastoma (U-87 MG-neuron). Blots were probed for PP2A B subunit and PPP2R5B and actin (loading control). Data represent three independent experiments (n = 3). Lower panel showed the densitometric quantification of western blot images. Values are presented as mean ± SEM. Statistical comparisons were performed using unpaired t-tests. Significance is indicated as: ns (not significant), *$P < 0.05$.

(C-D) Quantification of glioblastoma cell proliferation using Incucyte phase-contrast imaging. Selumetinib (MEK inhibitor) reduced proliferation in U-87 MG cells (C) but had no significant effect on NU-757 cells (D). Values are shown as mean ± SEM. Data were pooled from 4 independent cultures per condition, with 5 fields imaged per culture. **(E)** Barrier membrane blocks cell migration. Validation of the membrane's inhibitory efficacy was performed by comparing the non-invasive HMC3 microglial cell line with the invasive U-87 MG glioblastoma cell line, confirming selective prevention of tumor cell migration. (**F**) Migration assay of U-87 MG glioblastoma cells using Incucyte phase-contrast imaging. Selumetinib treatment inhibited both proliferation and migration of U-87 MG cells. Values are shown as mean ± SEM. Data were pooled from 3 independent cultures per condition, with 25 fields imaged per culture.

Figure S6. Effects of PP2A activation and combined MEK inhibition on glioblastoma and microglial cell proliferation.

(A-B) Quantification of cell proliferation using Incucyte phase-contrast imaging in U-87 MG glioblastoma cells (A) and HMC3 microglia (B) treated with DT-061 (PP2A activator) at increasing concentrations. DT-061 had minimal effects on both cell types, except at 10 µM, where it moderately reduced U-87 MG proliferation.

(C-D) Proliferation assays of U-87 MG glioblastoma (C) and HMC3 microglia (D) treated with a combination of Selumetinib (MEK inhibitor) and DT-061. The combination significantly suppressed U-87 MG cell proliferation, with partial inhibitory effects observed in HMC3 cells at 10 µM DT-061.

(E) Neurons treated with the specified inhibitors showed no signs of toxicity and maintained healthy morphology over a 4-day culture period.

Values are presented as mean ± SEM. Data were pooled from 4 independent cultures per condition, with 5 fields imaged per culture. Ctrl = untreated control; Sel = Selumetinib; DT = DT-061.

Figure S7. Glioblastoma cell viability and immunofluorescence controls. **(A)** Brightfield images of glioblastoma cells under different culture conditions. U-87MG cells were maintained in completed DMEM, and NU-757 cells in completed Neurobasal medium, as well as in neuronal maintenance medium and within the dual-interface co-culture system for 5 days. Images illustrate cell morphology and viability across these conditions. Scale bars = 20 μm

(B) Negative control images for immunofluorescence staining. Neuronal samples were processed without primary antibodies and stained with DAPI (nuclei, blue channel), Alexa Fluor 488 (green channel), and Alexa Fluor 647 (red channel) to confirm specificity of the staining. Scale bars = 20 μm

Table S1. Antibodies and dye

Antibody/Dye target	Species	Dilution		Company (Catalog #)
		IF	WB	
Anti-ERK1/2 (ERK-7D8)	Mouse	1:200	1:1000	Invitrogen (13-6200)
Anti-Phospho-ERK1 (T202/Y204)/ERK2 (T185/Y187)	Rabbit	1:200	1:1000	R&D Systems (MAB1018)
Anti -p38α MAPK	Rabbit	1:200	1:1000	Cell Signaling (#9218)
Anti-Phospho-p38 MAPK (Thr180/Tyr182)	Rabbit	1:100	1:1000	Cell Signaling (#9211)
Anti-Map2	Rabbit	1:200	1:1000	Abcam (ab32454)
Anti-MLK2	Rabbit	1:200	1:1000	Invitrogen PA5-75848
Anti-PPP2R5B	Rabbit	1:100	1:1000	Abcam (ab181023)
Rhodamine-Phalloidin	-	1:500	-	ThermoFisher (R415)
Alexa Fluor 488-phalloidin	-	1:500	-	ThermoFisher (A12379)
Anti-NMDAR2B	Mouse	-	1:1000	Abcam (ab93610)
Synaptophysin	Mouse	-	1:1000	Santa Cruz Biotechnology (sc-17750)
Anti-PSD95	Mouse	-	1:1000	Abcam (ab18258))
PP2A B Subunit (2G9)	Mouse	-	1:1000	Cell Signaling (mAb #5689)
Anti-PPP2R5B	Rabbit	-	1:1000	Antibodies-online (ABIN6243837)
Anti-β-Actin	Mouse	-	1:2000	Cell Signaling (#4967)
anti-Cyclophilin B (D1V5J)	Rabbit	-	1:2000	Cell Signaling (#43603)
Anti-rabbit IgG, HRP-linked Antibody	Rabbit	-	1:2000	Cell Signaling (#7074)
Anti-mouse IgG, HRP-linked Antibody	Mouse	-	1:2000	Cell Signaling (#7076)
Anti-β-Actin	Mouse	-	1:2000	Cell Signaling (#4967)
DAPI	-	1/5000		Invitrogen(D1306)
Alexa Flour 488	Mouse	1:200	-	Invitrogen (A32766)
Alexa Flour 647	Rabbit	1:200	-	Invitrogen (A-32733)
IRDye 680DR	Rabbit		1:10000	LI-COR (926-68073)
IRDye 800CW	Mouse		1:10000	LI-COR (926-32212)

Table S2. Neuronal medium components used for the study.

Media	Components	Company (Cat#)	Concentration	Period
Induction medium	Rock inhibitor	Tocris Bioscience (1254)	10µM	day 0
	DMEM/F12	Gibco (11320033)		day 0 - 3
	N2 supplement, 100×	Gibco (17502048)	1x	
	Non-essential amino acids (NEAA), 100×	Gibco (11140050)	1x	
	L-glutamine, 100× (or Gluta-MAX)	Gibco (25030081)	1x	
	Doxycycline	Sigma (D9891	2µg/ml	

Neuronal maintaining medium for cortical neurons	BrainPhys neuronal medium	STEMCELL Technologies (05790)		day 4 - 60
	Laminin	Gibco (23017015)	1µg/ml	
	B27 supplement, 50×	Gibco (17504044)	1x	
	BDNF	PeproTech (450-02)	10ng/ml	
	NT-3	PeproTech (450-03)	10ng/ml	
	Primocin	Invivogen (ant-pm-1)	50µg/ml	
