# Supplementary figures and images for: Dissecting acute neuronal responses to glioblastoma using a dual-interface human iPSC neuronal culture platform
Source: Acta Neuropathol Commun. 2026 May 16;14:149. doi: 10.1186/s40478-026-02312-z (PMC13371652; doi:10.1186/s40478-026-02312-z)

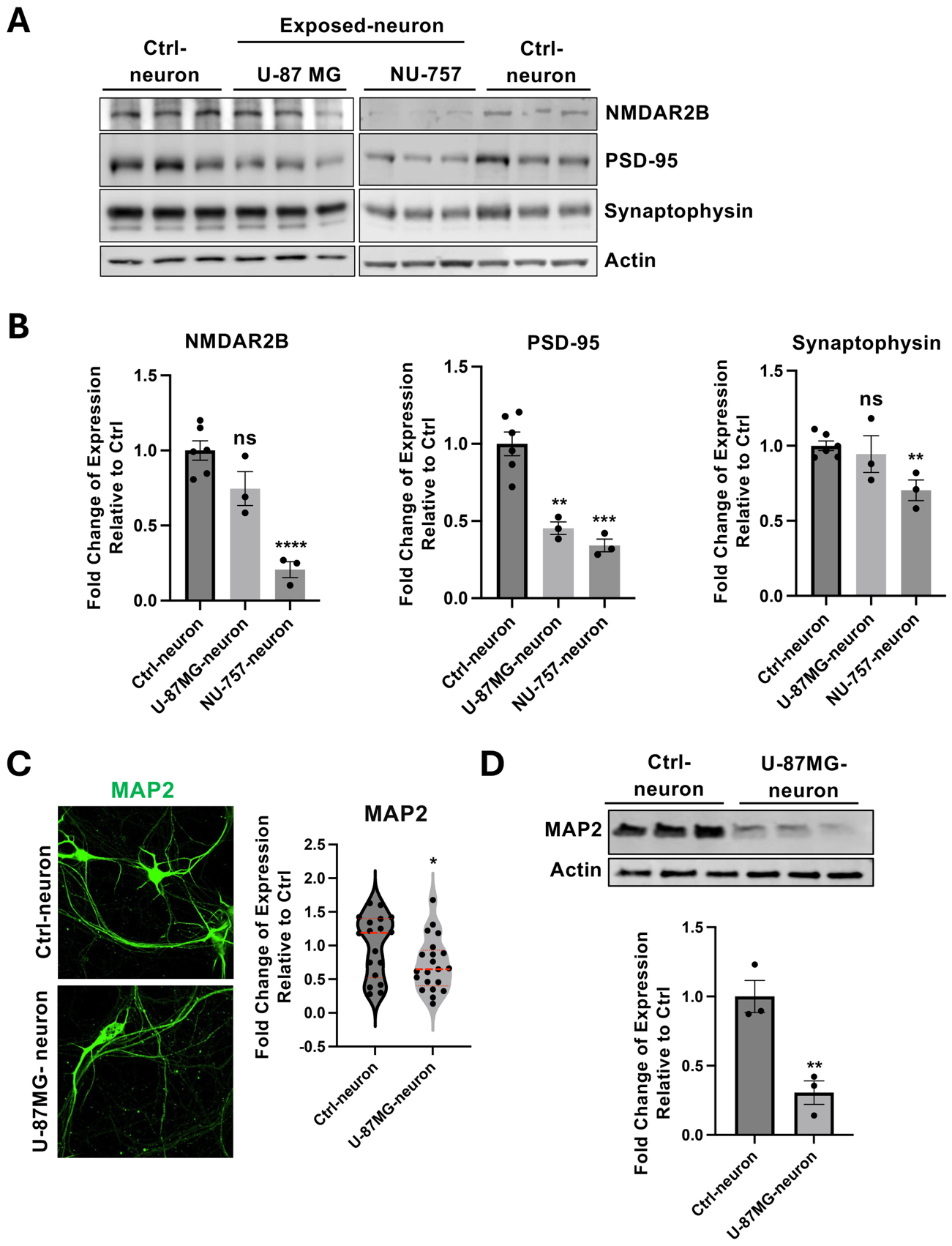


Figure S1.


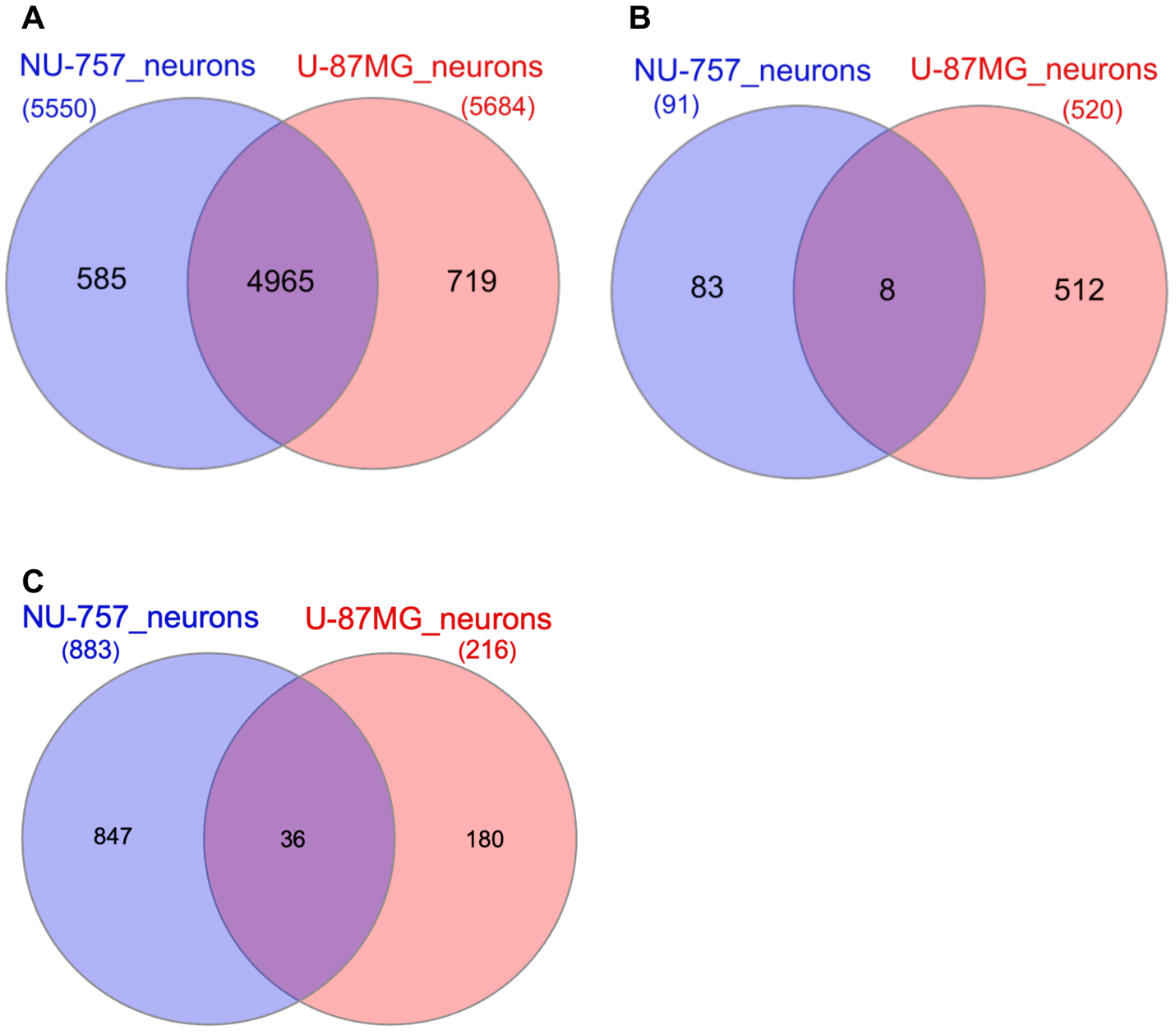


Figure S2.


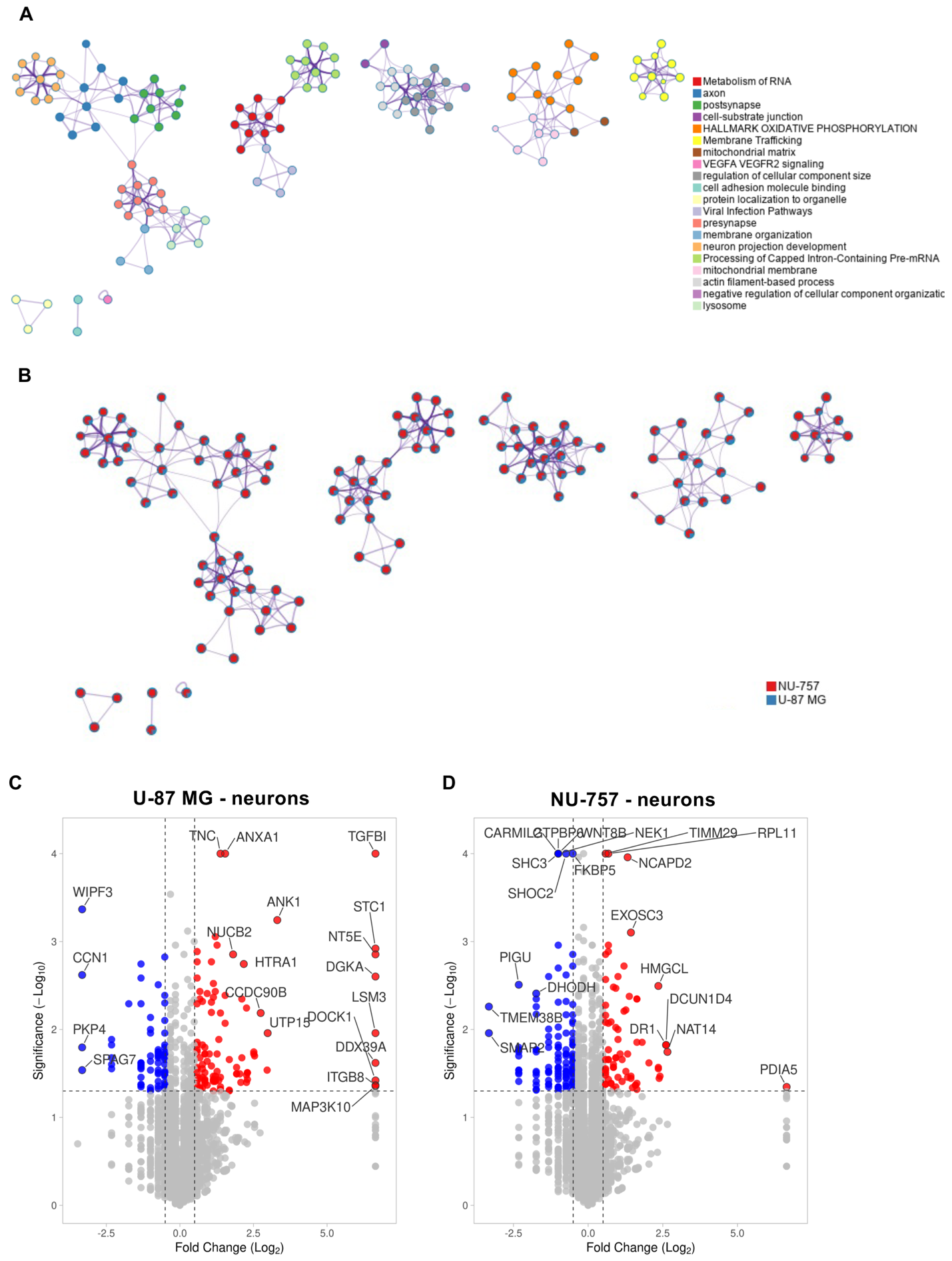


Figure S3.


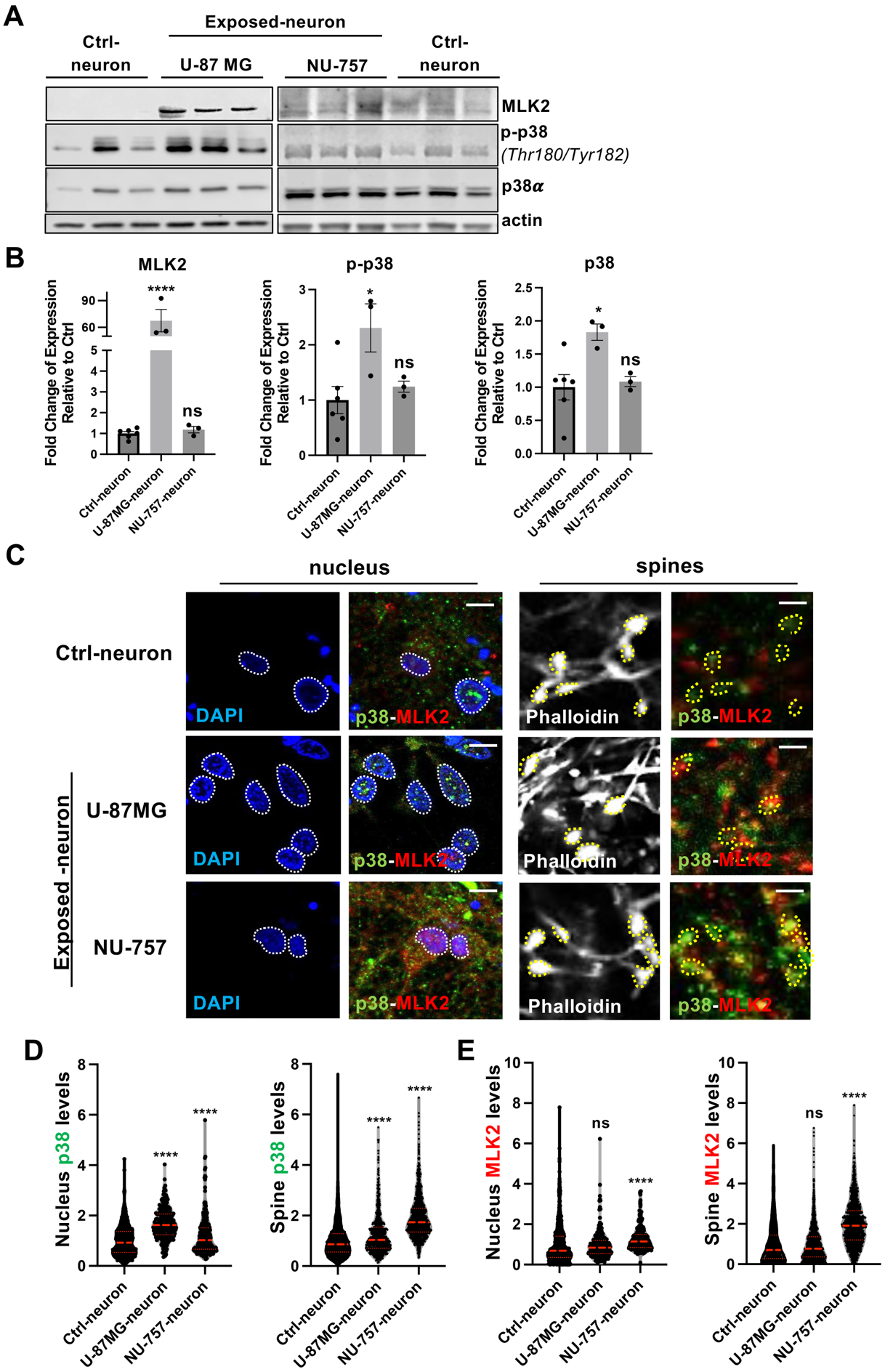


Figure S4.


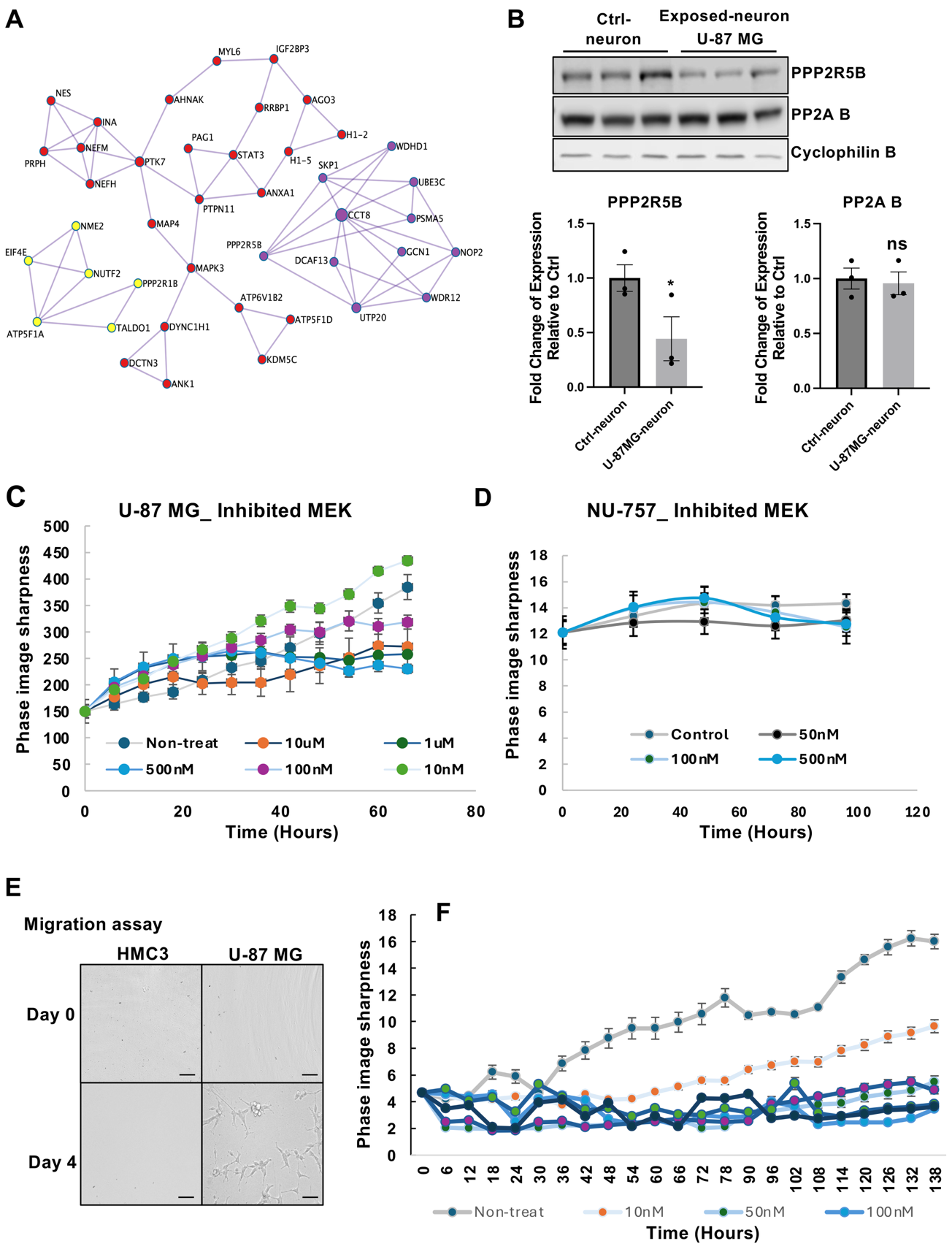


Figure S5.


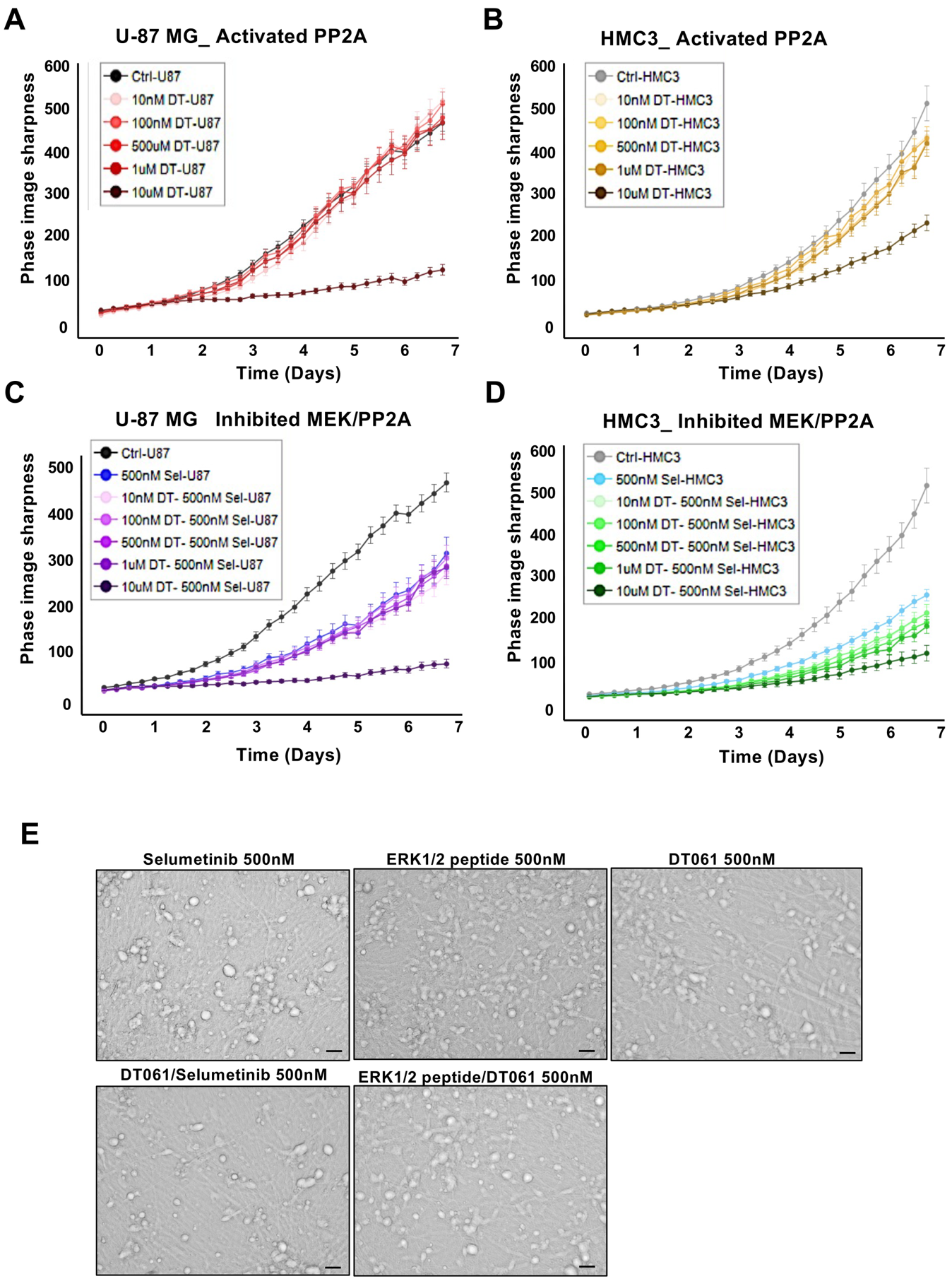


Figure S6.


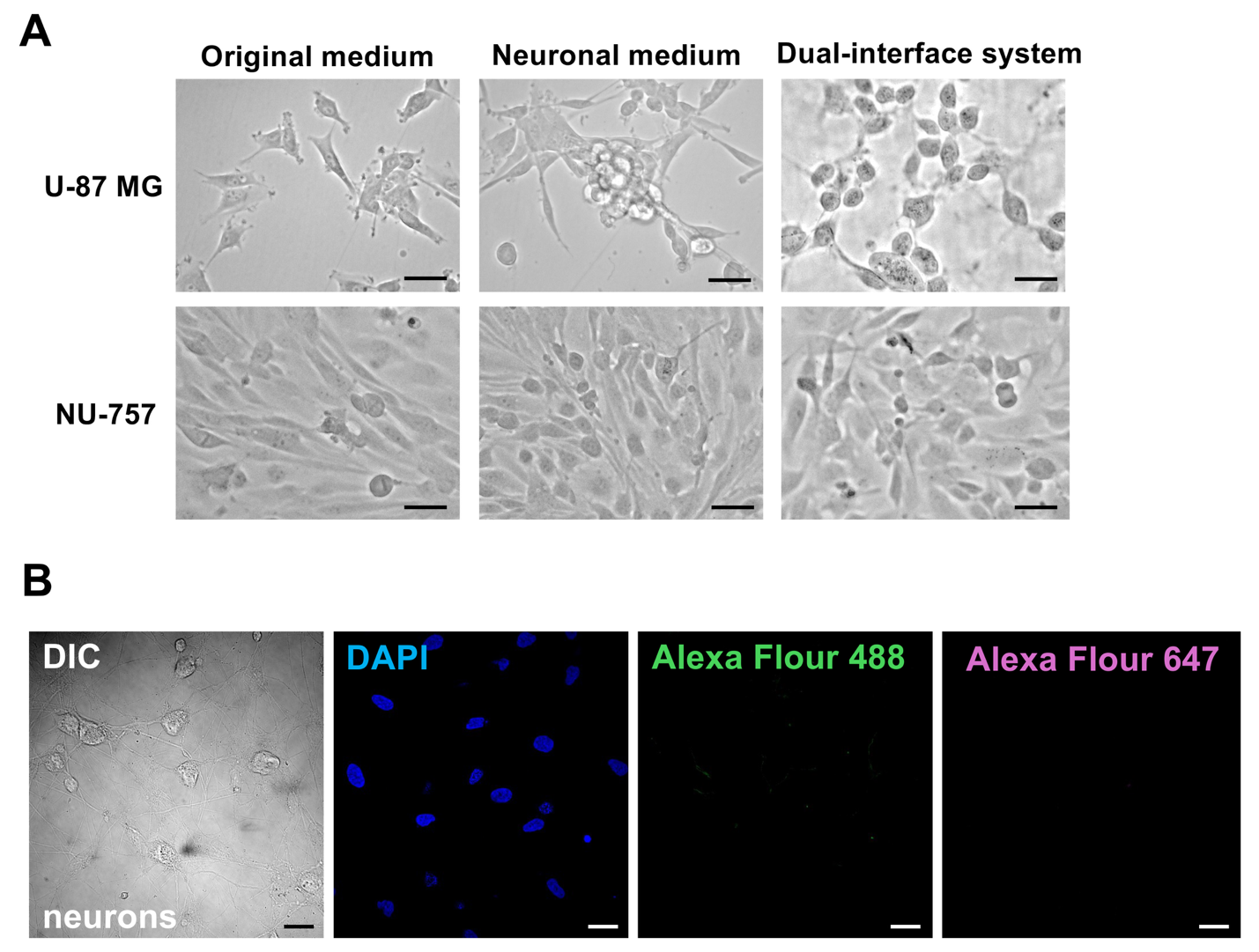


Figure S7.

Supplement: Supplementary file 3 — Supplementary Material 3 [file 40478_2026_2312_MOESM3_ESM.docx]
